# Serial processing of stimulus identity and shift readiness predictions

**DOI:** 10.3758/s13414-025-03137-z

**Published:** 2025-08-05

**Authors:** Anthony W. Sali, Emily E. Oor

**Affiliations:** Department of Psychology, Wake Forest University, 1834 Wake Forest Road, Winston-Salem, NC 27109 USA

**Keywords:** Cognitive and attentional control, Attention and executive control, Attention: space-based

## Abstract

As an individual’s goals change, they must flexibly shift the focus of attention. In real-world scenarios, multiple stimuli, each with different likelihoods, can signal that it is appropriate to shift or to hold attention on a moment-by-moment basis. In the current study, we independently manipulated the likelihood of shifting attention and the likelihood of receiving particular cue stimuli by presenting multiple shift- and hold-attention cues to dissociate the behavioral costs associated with violations of shift readiness from stimulus identity predictions. After excluding trials with exact cue stimulus repetitions—where response times were significantly shorter than for other trials—we observed additive updating costs for shift and stimulus identity likelihood prediction errors across two experiments. Together, our results suggest that when cue stimuli do not consecutively repeat, the processes of updating attention-shifting readiness and stimulus identity are best explained by a serial processing architecture. The data, materials, and analysis code for this experiment are available online (https://osf.io/zdtvc/).

**Supplementary Information:**

The online version contains supplementary material available at 10.3758/s13414-025-03137-z.

An important facet of attentional control is the ability to update the focus of attention in response to changing goals. Individuals regularly experience fluctuations in their readiness to shift attention, referred to here as attentional flexibility, such that the behavioral cost associated with shifting varies over time (Sali et al., 2016). In addition to intrinsic spontaneous fluctuations in attentional flexibility, individuals can harness learned expectations to adjust their moment-by-moment shift readiness to best meet current environmental demands (Sali et al., 2015). Previous tests of learned attentional flexibility have often confounded shift and stimulus identity predictions such that a prediction to shift coincides with a prediction to receive a particular stimulus. Real-world scenarios have much greater complexity, offering multiple stimuli that could signal individuals to shift or hold attention with different likelihoods of occurring. In the current study, we manipulated shifting expectations and cue stimulus identity expectations independently, allowing us to dissociate cue type prediction errors (e.g., expecting to shift attention but receiving a hold cue) from cue stimulus identity predictions errors (e.g., expecting to see cue stimulus A, but receiving cue stimulus B).

Growing evidence suggests that reinforcement learning (RL) serves as a mechanism by which individuals make and update predictions regarding optimal states of attentional control. Even in the absence of overt rewards, accurate performance is intrinsically rewarding, and ongoing adjustments in control states allow individuals to best prepare for upcoming cognitive demands (Braem & Egner, 2018). If an outcome matches an individual’s expectations, there is little need for updating. However, if an outcome is surprising and violates these expectations, the magnitude and nature of the violation is used to update the prediction (Sutton & Barto, 1998). This form of RL successfully accounts for trial-by-trial performance in diverse areas of cognitive control such as task-switching readiness (Sali et al., 2024), response conflict control (Chiu & Egner, 2017; Chiu et al., 2017), and spatial attentional flexibility (Sali & Egner, 2020).

In addition to tracking the likelihood of demands on attentional control, individuals also track stimulus identity likelihoods. The detection of deviant stimuli in oddball paradigms is associated with increased response time, reflecting a violation of expectations (Vossel et al., 2009). Furthermore, there is partial overlap in the frontal, parietal, and temporal cortical regions that are implicated in the signaling of both attention shifting readiness prediction errors as well as nonspatial stimulus surprise, suggesting that there may be some interaction between these two forms of violation detection (Bledowski et al., 2004; Downar et al., 2000; Marois et al., 2000; Vossel et al., 2009). Models of predictive coding stipulate that sensory information is compared with prediction-derived template representations in order to resolve perceptual ambiguity (Bar, 2007; Summerfield et al., 2006). Central to this theory is the idea that distinct neural populations throughout the visual system represent sensory predictions and prediction errors (Rao & Ballard, 1999; for a review, see Walsh et al., 2020).

Although the interaction of cue stimulus and shift likelihoods is not well understood in the context of spatial attentional control, the task-switching literature offers insights regarding the isolation of cue encoding processes from the act of switching itself. In a traditional task-switching paradigm, a single cue is associated with each task, meaning that task switches, but never task repeats, are associated with a trial-by-trial change in the cue stimulus. To dissociate cue switching from task switching, researchers have adopted designs in which there are multiple cues per task, such that a change in the cue stimulus could signal both a task repetition and a task switch (e.g., Logan & Bundesen, 2003; Mayr & Kliegl, 2003). When separating cue- and task-switching costs in this way, much of the behavioral cost that was traditionally associated with task-switching could be attributed to a change in the cue stimulus. However, interpreting these findings is not straightforward. Logan and Bundesen (2003) argued that participants in a task-switching experiment encode the cue and the stimulus and then respond based on the compound, thus incurring behavioral costs due to a change in stimulus instead of a change in task. More recently, the benefits associated with cue repetitions have been linked to both the repetition of the same cue stimulus form, referred to as perceptual priming, as well as repetition at the conceptual level, such as the cue “D” following the cue “d” (Schneider, 2016). Conversely, Mayr and Kliegl (2003) claimed that cue-switch costs reflected the need to load the task set from long-term memory to working memory and that this cost was added to a relatively smaller cost associated with applying the task set to the stimulus. To address the limitation that a cue repetition was always associated with a task repetition, Forstmann et al. (2007) adopted transition cues that signaled participants to switch or repeat task, rather than cueing the exact task itself. Under these conditions, participants produced both cue-switch costs (slower responses on trials with a cue switch than a cue repeat) when the task repeated as well as cue-switch benefits (faster responses on trials with a cue switch than a cue repeat) when there was a task-switch, suggesting that traditional switch costs may not merely reflect cue priming (Forstmann et al., 2007). Taken together, a combination of cue and task transition histories are likely to shape moment-by-moment performance (Schneider & Logan, 2007).

Manipulations of task-switching likelihood have also shed greater light on the existence of switch costs that extend beyond cue stimulus processing. Although switch costs are diminished and performance may be more influenced by cue priming effects when the probability of switching is at least moderately high, robust switch costs that are dissociable from cue priming are observed when the probability of switching is low (Monsell & Mizon, 2006). While this switching probability effect could be interpreted as a modulation of switch readiness, it could also stem from participants learning specific high-probability cue–cue transitions (Schneider & Logan, 2006). However, when cue transitions and switch transitions were both manipulated, individuals demonstrated larger cue-switch costs and smaller task-switch costs as the probability of switching tasks increased, but there was no reliable effect associated with high probability cue–cue transitions (Mayr, 2006). Individuals seem to engage in ongoing learning regarding the statistical structure of their environment, adjusting their switching readiness to meet moment-by-moment changes in the anticipated demands (Crump & Logan, 2010; Dreisbach & Haider, 2006; Leboe et al., 2008). In the current study, we adapt the multiple transition cue design from the task-switching literature to manipulate both shift and stimulus identity likelihoods in a spatial attentional control task.

Despite evidence that individuals rely on both shifting likelihood and stimulus identity predictions to guide behavior, to our knowledge these two key processes have not been examined together in the context of spatial attentional control. We tested whether shift likelihood and stimulus identity prediction updating are carried out serially, incur compounding costs, or may proceed at least partially in parallel. As in previous tests of spatial attentional flexibility (Sali et al., 2015, 2016, 2022; Sali & Egner, 2020), participants completed a rapid serial visual presentation (RSVP) task in which they monitored a stream of alphanumeric characters for the appearance of a letter cue, which signaled them to either hold their attention at the location of the cue or to shift attention to the opposite location. Importantly, as in previous studies (e.g., Forstmann et al., 2007), we employed two shift and two hold cues, allowing us to independently manipulate shift and stimulus identity likelihoods. Stimulus identity likelihoods varied across participants while shift likelihood varied on a block-by-block basis for each participant. We hypothesized that violations of both shift readiness and stimulus identity predictions would be associated with slower response times (RT). Our design could yield three potential patterns of behavioral results, each of which would indicate the degree to which shift readiness and stimulus identity updating interfere with one another (Townsend, 1990).

First, it is possible that we would observe additive costs indicating that updates of spatial attentional flexibility and identity predictions must proceed in serial. This finding would indicate that the component processes associated with updating shift readiness or stimulus identity predictions must complete before the remaining updating operation (either shift readiness or stimulus identity) may begin. For example, one possibility is that stimulus identity predictions are fully processed first since identity is needed to interpret the cue’s meaning. Under this interpretation, shift readiness and stimulus identity updating reflect dissociable mechanisms that are carried out with a serial dependence. Alternatively, there may be a shared mechanism that is needed for both forms of updating but that can only process one update at a time. This scenario would create a bottleneck that forces one updating operation to wait until the other operation is completed (Pashler, 1994; but see below for a discussion of how a bottleneck might not always lead to additive costs).

Second, we could observe a subadditive interaction. This would be the case if separable mechanisms are responsible for shift readiness and stimulus identity prediction updating and these mechanisms proceed, at least partially, in parallel. In this scenario, the total time needed for both updating operations to occur should be less than the sum of the two processes in isolation if both processes must be exhaustively completed (see Kessler, 2017, for a similar logic). One updating operation may begin before the other is completed and therefore does not fully depend on the output of the first operation. For example, it is possible that stimulus identity updating only needs to proceed through a certain stage prior to the initiation of shift readiness updating, with a period during which both processes are executed in parallel. This account does not rule out the existence of a processing bottleneck. One or more component processes for shift readiness and stimulus identity updating may require a shared mechanism that is limited to a single operation at a time. However, if at least two processes are initiated in parallel, the amount of time that the bottleneck is empty is minimized. Therefore, a processing bottleneck will lead to additive costs if the second-initiated updating operation has no prebottleneck processes that may begin while the first operation is still running. However, a processing bottleneck will yield a subadditive interaction if some prebottleneck subprocesses proceed in parallel with the first-initiated updating operation, thus reducing the total processing time (Pashler, 1994). Alternatively, we may also observe a subadditive interaction if completing one form of prediction updating primes or speeds the rate at which a subsequent update is executed such that completion of one update facilitates the next.

Third, it is possible that we would observe a superadditive interaction of the two processes, indicating that completing one form of updating interferes with the other. For example, if shift readiness and stimuli identity updating are carried out in serial but share a supervisory process that must itself be shifted, the total time needed to complete both updates together would exceed the sum of each carried out independently. An example of this interference could come from a shared control process that regulates predictions and must shift from the first operation carried out to the second, thus incurring an additional shift cost on top of the time needed to perform each independent updating operation.

Given our design, we predicted that the RT slowing associated with violations of shift readiness predictions would result in larger behavioral shift costs for low shift likelihood blocks than for high shift likelihood blocks (e.g., Sali et al., 2015). Consequently, evidence of stimulus identity updating would be indicated by a significant slowing on unexpected stimulus identity trials relative to expected stimulus identity trials, while shift readiness updating would lead to a significant cue type (shift vs. hold) by block-wide shift likelihood (high shift likelihood vs. low shift likelihood) interaction. Thus, to test the interaction of shift readiness updating with stimulus identity updating, we examine the three-way interaction of cue type (shift vs. hold), block-wide shift likelihood, and stimulus identity likelihood. Additive costs would therefore result in no significant three-way interaction indicating that the change in attention shifting costs across high and low shift likelihood blocks is the same for high and low likelihood stimuli. A subadditive interaction would be indicated by block-wide attention shifting cost differences that are smaller for low likelihood stimulus identities than for high likelihood stimulus identities, favoring a parallel processing interpretation. Conversely, a superadditive interaction would be indicated by a difference in shift costs across blocks that was larger for low than high likelihood stimulus identities, as would be expected if there is interference between updating operations.

## Experiment 1

In Experiment 1, we manipulated both shift and cue stimulus identity likelihoods in an RSVP attention-shifting task. By extending this paradigm to include four cues (two shift and two hold), we factorially controlled the likelihood of shifting across alternating blocks of trials and the likelihood of receiving a particular cue between subjects. The RSVP task affords several advantages for studying the interaction of shift and stimulus identity statistical learning. First, the task requires participants to hold their attention at a spatial location as they wait for a cue stimulus to appear among distractor filler stimuli. This requirement allowed us to precisely control where attention must be directed lending to our ability to dissociate cue identity and shift likelihood predictions in a spatially specific manner. Second, this setup also mirrored real-world scenarios in which individuals maintain focus on goal-relevant locations—such as watching for a bus arrival at a busy terminal—while filtering out frequent, competing distractors that do not signal a need to reorient. Third, by requiring participants to make a parity judgment of the first digit they detected at the to-be-attended location after the onset of the cue, we acquired a trial-by-trial measure of shift readiness indexed by the RT. Given our design, the three-way interaction of cue type (shift vs. hold), block-wide shift likelihood (high vs. low), and stimulus identity likelihood (high vs. low) would indicate whether shift and stimulus violations can be processed at least partially in parallel or are constrained with a serial architecture.

### Method

#### Participants

Fifty-two individuals (36 men, 15 women, one did not wish to reply) ranging in age from 19 to 42 years (*M* = 32.35 years, *SD* = 5.75) participated online through the website Prolific (https://www.prolific.com) in exchange for $12 compensation. Participants agreed to participate with a virtual consent form and all procedures were approved by the Wake Forest University Institutional Review Board in accordance with the 1964 Declaration of Helsinki. All participants self-reported normal or corrected-to-normal vision with no colorblindness, had a Prolific approval rating between 90 and 100, were currently in the USA, and had previously completed at least 100 submissions on Prolific. Of the individuals who completed the study, two were excluded from all analyses for having overall behavioral accuracies that were less than 70%.^1^ One participant reported that their keyboard disconnected during the fifth block of trials, leading to a reduced number of trials with a correct response in one probability context relative to the other. However, given that the participant’s overall accuracy across all runs remained over 70%, we elected to include their data from all runs in all analyses.

We conducted a power analysis using the R package *Superpower* (Lakens & Caldwell, 2021), to simulate the number of participants needed to reach 95% power for the interaction of cue type (shift vs. hold) and block-wide shift likelihood (low vs. high) according to the means and correlations among conditions from a previous test of learned attentional flexibility (see Sali et al., 2015, Experiment 3). For this simulation we set the standard deviation of each condition to be equal to the greatest single condition standard deviation observed in the previous study. This simulation indicated that we surpassed 95% power with 5 participants. However, to focus on the primary comparison—the three-way interaction of cue type, block-wide shift likelihood, and stimulus identity likelihood—and to mitigate the increased noise from online data collection, we increased the sample size by approximately tenfold.

#### Stimuli

Stimulus presentation was controlled by PsychoJs (Version 2023.1.3) as implemented in PsychoPy (Peirce et al., 2019) and run within the participant’s web browser. The task was hosted on Pavlovia (https://pavlovia.org). Since we did not have control over the participant’s viewing distance, we do not report stimulus visual angles below. However, participants did complete a brief task in which they resized a credit card on their display to match the size of a real credit card to compute a stimulus scale factor. This procedure was repeated at least two times for each participant until the two scaling estimates in both the *x* and *y* dimensions deviated by no more than 5%. This procedure approximately equated the size of stimuli on each participant’s screen.

Participants viewed displays that consisted of two RSVP streams of alphanumeric characters positioned to the left and right of the center of the display. All stimuli were presented in white against a gray background. The streams consisted of digits ranging from 1 to 8 and the letters “S,” “C,” “H,” “K.” There were never any numbers or letters repeated across consecutive frames. Each frame of the display was presented for 250 ms with no gap between frames.

#### Procedure

**Fig. 1:**
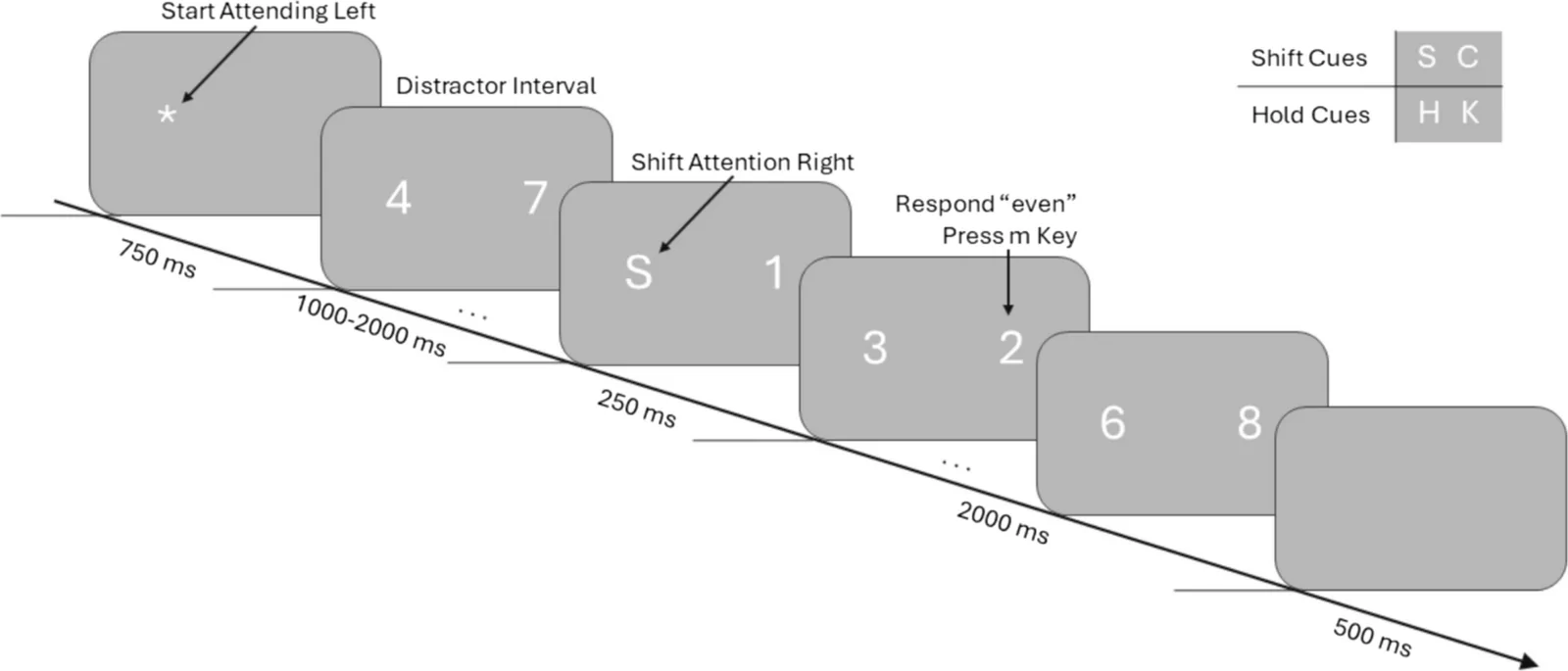
Experimental task. Participants monitored one out of two task-specific peripheral RSVP (rapid serial visual presentation) streams for a visual cue. Letter cues directed participants to either shift/change (“S” and “C”) attention to the opposite stream or hold/keep (“H” and “K”) attention on the current stream. Following each signal, subjects quickly determined the parity of the stimuli appearing at the cued location, pressing the “m” key for even numbers and the “z” key for odd numbers

Each trial began with an asterisk that flashed at the to-be-attended region for 750 ms (Fig. 1). Next, the RSVP streams appeared. For an interval of 1,000 ms, 1,500 ms, or 2,000 ms, referred to below as the distractor interval, randomly generated numbers appeared at each of the streams. After this delay, a letter cue appeared at the to-be-attended location that instructed the participant to either hold their attention at the location of the cue or shift their attention to the opposite location. The letters “H” and “K” always signaled participants to hold (“Hold” and “Keep”), while the letters “S” and “C” signaled participants to shift (“Shift” and “Change”). Immediately following the onset of the letter cue, the numbers generated at the cued location were either all odd or all even for a 2,000-ms response window. Participants were instructed to respond based on the first digit that they identified at the cued location, specifically judging whether the digit was even or odd, such that response times (RT) served as an indicator of shift readiness (e.g., Sali et al., 2015). During the response window, participants pressed the “z” key of their keyboard if they detected odd digits and the “m” key if they detected even digits. The streams continued for the entire response window regardless of whether the participant made a button press. For each participant, we randomly assigned half of the trials to have odd target stimuli and half to have even target stimuli. After the response window, there was a 500-ms blank interval. The asterisk would then appear at the last attended location to begin a new trial.

As in previous studies, we manipulated the likelihood of receiving shift and hold attention cues across blocks of 80 trials each. Each block contained either 75% shift cues (high shift likelihood blocks) or 25% shift cues (low shift likelihood blocks) and the high and low shift likelihood blocks alternated. However, unlike previous studies, we also manipulated the likelihood of the individual letter stimuli. Specifically, for each participant, one of the shift cues (“S” or “C”) appeared on 75% of shift trials, and one of the hold cues (“H” or “K”) appeared on 75% of hold trials in both high and low shift likelihood blocks. Thus, shift likelihood varied on a block-by-block basis while stimulus identity likelihood varied across participants. We counterbalanced the stimulus identity probabilities and whether participants began with a high or low shift likelihood block, yielding a total of eight combinations. In our final sample, the number of participants in each of these eight conditions ranged from four to eight (*M* = 6.25, *SD* = 1.16).

Participants completed a scaffolded practice session, during which they first completed the task at a slower pace (each frame presented for 500 ms) and with cue stimuli appearing in red. They then completed trials at the pace of the main task with the cue stimuli still appearing in red prior to a final practice session that was identical to the main task. Each cue stimulus was equally likely during the practice session and participants completed each practice phase in blocks of eight trials until they received an overall accuracy of at least 70%, at which point they could move on to the next phase. Following completion of the practice, participants completed six blocks consisting of 80 trials each of the main task. We collected demographic information with a Qualtrics survey.

#### Data analysis and availability

Our primary analyses focused on RT for trials during which the participant made the correct parity judgment. Given the large differences in the number of trials per condition, data trimming procedures must be carefully selected to not artificially trim more data from some conditions than others. Due to recent evidence that warns against trimming procedures with variable cutoffs based on factors such as standard deviations (Miller, 2023) we elected to trim RTs that were less than 200 ms only, as these would reflect responses that were made before the participant could have accurately responded to the target stimuli. This procedure yielded a reduction of less than 1% of all trials with an accurate response. The offset of the RSVP streams at the end of each trial marked the upper limit for RTs. Given that our primary aim was to test the interaction of shift readiness and stimulus identity prediction updating, and that a null result would be suggestive of a serial processing relationship, we used both frequentist and Bayesian repeated-measures analyses of variance (ANOVAs) for the primary RT analyses. While we focus on RTs, because they are most diagnostic of processing architecture given our design, we also report behavioral accuracies for completeness and to rule out the possibility of speed–accuracy trade-offs. For all accuracy analyses reported, we included all trials regardless of the magnitude of the RT because removing trials with outlier RTs would potentially inflate accuracies and obscure differences in error rates across conditions.

All data, stimulus presentation code, and analysis code for Experiments 1 and 2 are available online (https://osf.io/zdtvc/). We analyzed the data from both experiments using MATLAB R2023a (Version 9.14.0.2286388) and R (Version 4.1) with the following packages: *tidyverse* (Wickham et al., 2019), *afex* (Singmann et al., 2024), *Superpower* (Lakens & Caldwell, 2021), and *cowplot* (Wilke, 2024). We used JASP (Version 0.19.1) for Bayesian repeated-measures ANOVAs.

### Results

**Fig. 2:**
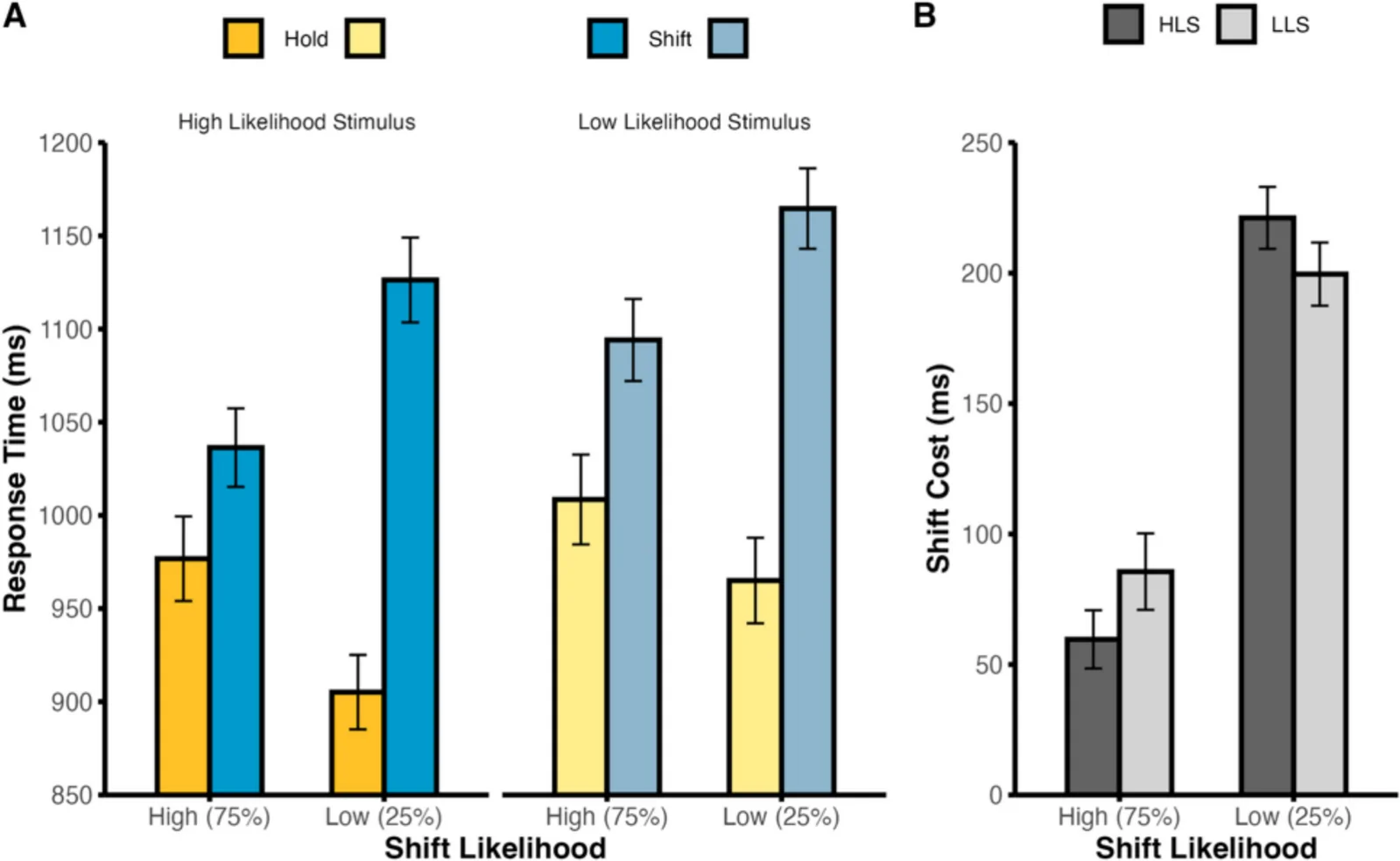
Behavioral response times on trials with a high likelihood stimulus (**A**; left) versus trials with a low likelihood stimulus (**A**; right) in Experiment 1. Comparison of the shift costs between high and low shift likelihood blocks for each stimulus identity likelihood condition (**B**). Dark-gray bars represent the high likelihood stimulus (HLS) condition, while light-gray bars represent the low likelihood stimulus condition (LLS). Error bars denote ±1 between-subjects *SEM*. (Color figure online)

We first analyzed RTs with a repeated-measures ANOVA, with the factors cue type (hold vs. shift), block-wide shift likelihood, (25% shift block vs. 75% shift block), and stimulus identity likelihood (25% occurring letter vs. 75% occurring letter). As predicted, there were costs associated with both shifting attention and receiving an unexpected stimulus, such that participants had slower RTs on shift trials than on hold trials, *F*(1,49) = 278.57, *p* < .001, η_p_^2^ = .850, and were slower to respond on low likelihood stimulus trials than on high likelihood stimulus trials, *F*(1,49) = 41.09, *p* < .001, η_p_^2^ = .456 (see Fig. 2A). There was no significant main effect of shift likelihood, *F*(1,49) = 1.77, *p* = .189, η_p_^2^ = .035. As in previous studies, the interaction of cue type and shift likelihood reached significance, *F*(1,49) = 147.55, *p* < .001, η_p_^2^ = .751, such that the cost associated with shifting attention was larger in low shift likelihood blocks than in high shift likelihood blocks. Most interestingly, the three-way interaction reached significance, *F*(1,49) = 7.96, *p* = .007, η_p_^2^ = .140 (see Fig. 2B). The shift cost modulation between low and high shift likelihood blocks was larger for high probability stimuli than for low probability stimuli, indicating a subadditive relationship between shift likelihood and stimulus identity likelihood prediction updating. Follow-up ANOVAs revealed significant shift cost modulations across the two shift likelihoods for both high, *F*(1,49) = 124.30, *p* < .001, η_p_^2^ = .717, and low, *F*(1,49) = 68.68, *p* < .001, η_p_^2^ = .584, probability stimuli. The remaining two-way interactions of the main ANOVA did not reach significance (*F* values < 0.28, *p* values > .599).

We next analyzed the RT data with a Bayesian repeated-measures ANOVA to estimate the evidence in favor of or against a three-way interaction of cue type, block-wide shift likelihood, and stimulus identity likelihood. Here, and in all future analyses, we used uniform priors and set the number of samples determining numerical accuracy to 100,000. The best performing model contained regressors for cue type (shift vs. hold), shift likelihood (high vs. low), stimulus identity likelihood (high vs. low), and the interaction of cue type by shift likelihood: BF_10_ = 3.591×10^37^ relative to the null model, which accounted for participant and random slopes (see Supplemental Table 1 for full model comparison).

We also obtained the inclusion Bayes factors for each of the main effects and interactions in our design (see Table 1). Here, and in all subsequent Bayesian analyses of effects, we compute BF_inc_ for each effect by comparing models with the effect to only equivalent models that are stripped of the effect of interest. Higher-order interactions were excluded. There was decisive evidence in favor of the interaction of cue type and block-wide shift likelihood, BF_inc_ = 3.782×10^13^, and substantial evidence against the interaction of block-wide shift likelihood and stimulus identity likelihood, BF_inc_ = 0.187, as well as cue type and stimulus identity likelihood, BF_inc_ = 0.208. Most interestingly, there was strong evidence in favor of a subadditive interaction of shift readiness and stimulus identity updating, BF_inc_ = 13.888.

**Table 1:** Analysis of effects for all trials in Experiment 1

Effect	BF_inc_
CueType	8.266×10^18^
ShiftLike	0.467
StimLike	254,862.341
CueType × ShiftLike	3.782×10^13^
CueType × StimLike	0.208
ShiftLike × StimLike	0.187
CueType × ShiftLike × StimLike	13.888

*Note.* CueType denotes shift vs. hold, while ShiftLike and StimLike denote the shift and stimulus identity likelihoods, respectively. Models are only compared with equivalent models with higher-order interactions excluded

To rule out the possibility of a speed–accuracy trade-off, we next ran an ANOVA on behavioral accuracies. As with RTs, there was a significant main effect of cue type, *F*(1,49) = 7.56, *p* = .008, η_p_^2^ = .134, such that participants were more accurate on hold attention trials than on shift attention trials (see Table 2). A significant three-way interaction paralleled the RT results, *F*(1,49) = 4.44, *p* = .040, η_p_^2^ = .083. No other main effects or interactions from the main ANOVA reached significance (*F* values < 1.60, *p* values > .213).

**Table 2:** Behavioral accuracies across all trials in Experiment 1

	Low stimulus identity likelihood		High stimulus identity likelihood	
	Low shift likelihood	High shift likelihood	Low shift likelihood	High shift likelihood
Hold attention	92.09 (8.97)	92.00 (10.69)	93.20 (7.78)	91.38 (8.45)
Shift attention	90.13 (12.94)	89.87 (10.98)	89.73 (11.67)	90.67 (10.36)

*Note. *Mean percentage accuracies (*SD*).

**Fig. 3:**
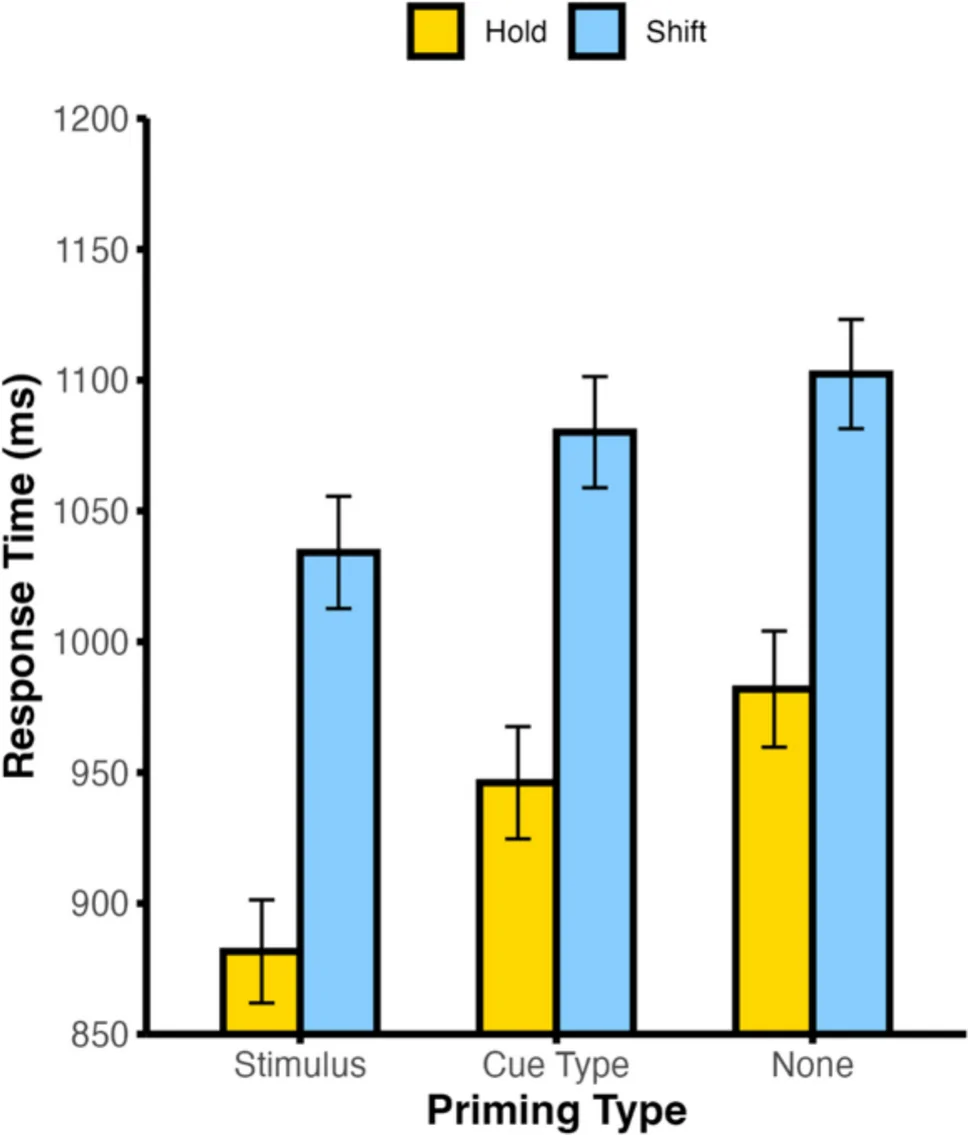
Comparison of trial-by-trial priming effects on average response times in Experiment 1. Error bars denote ±1 between-subjects *SEM*. (Color figure online)

Although the results described thus far are suggestive of a subadditive relationship between shift readiness and stimulus identity prediction updating, they do not account for differences in the likelihood of trial-by-trial priming across conditions. To isolate the impact of cue repetition and stimulus repetition priming, we excluded the first trial of each block and divided the remaining trials according to whether the cue received on trial *n* was the same type (shift vs. hold) and/or stimulus identity (“S” vs. “C”) as the cue received on trial *n* − 1. This grouping according to whether the cue type and the stimulus identity repeated across consecutive trials, yielded trials with identical cues across consecutive trials (stimulus priming: e.g., “S” followed by “S”), a stimulus identity change with repeating cue type (cue type priming: e.g., “H” followed by “K”), and neither a stimulus identity nor cue type repeat (none: e.g., “S” followed by “K”). We further divided the data based on whether each trial required an attention shift or hold and subjected these data to a repeated-measures ANOVA, with the factors cue type (shift vs. hold) and priming condition (stimulus, cue type, or none). As above, RTs were slower for shift trials than for hold trials, *F*(1,49) = 256.87, *p* < .001, η_p_^2^ = .840. Both the main effect of repetition condition, *F*(2,98) = 90.52, *p* < .001, η_p_^2^ = .649, and the interaction of cue type by repetition condition, *F*(2,98) = 5.11, *p* = .008, η_p_^2^ = .094, also reached significance (see Fig. 3).

To better understand these patterns, we conducted a series of pairwise comparisons for shift and hold trials separately. To control the family-wise error rate, we Bonferroni corrected the following six comparisons by setting the critical alpha at *p* = .008. For hold trials, RTs were significantly longer for no priming trials relative to both cue type priming, *t*(49) = 4.47, *p* < .001, *d*_z_ = 0.633, and stimulus identity priming, *t*(49) = 11.70, *p* < .001, *d*_z_ = 1.655, trials. There was also a significant difference between cue type priming and stimulus identity priming hold trials, *t*(49) = 9.42, *p* < .001, *d*_z_ = 1.333. For shift trials, the difference between no priming and cue type priming trials did not reach significance after correcting for multiple comparisons, *t*(49) = 2.41, *p* = .020, *d*_z_ = 0.340. However, there were significant differences between cue type priming and stimulus identity priming trials, *t*(49) = 6.32, *p* < .001, *d*_z_ = 0.894, and between no priming and stimulus identity priming trials, *t*(49) = 8.01, *p* < .001, *d*_z_ = 1.133. Thus, our results suggest that for both shift and hold trials, a repetition of both the cue type and stimulus identity (an exact cue stimulus repetition across trials) yielded a significant reduction in RTs relative to a repetition of cue type alone.

**Fig. 4:**
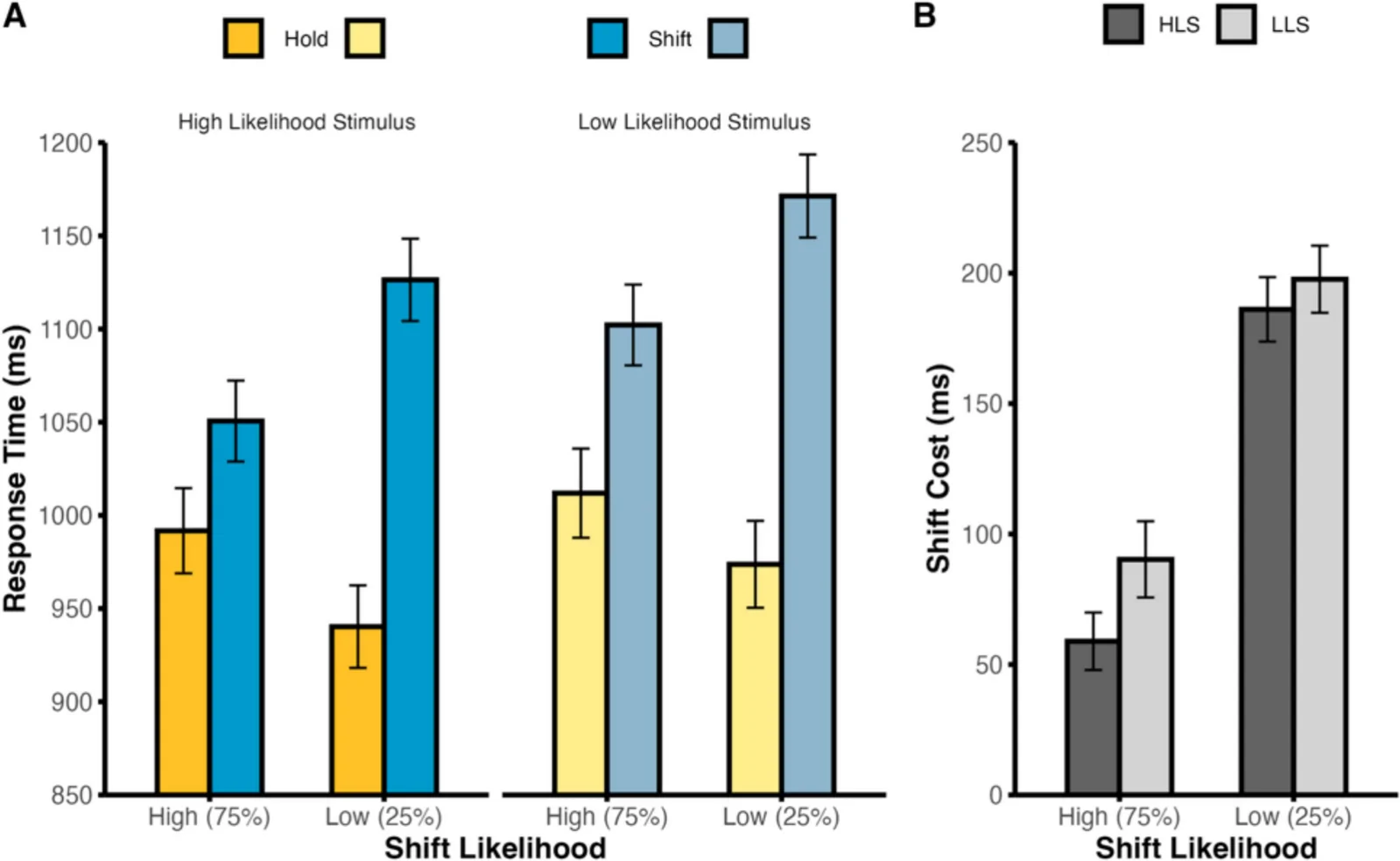
Behavioral response time analysis excluding trials containing exact repetitions of stimulus identity in Experiment 1. Behavioral response times for trials with a high likelihood stimulus (**A**; left) versus trials with a low likelihood stimulus (**A**; right). Comparison of the shift costs between high and low shift likelihood blocks for each stimulus identity likelihood condition (**B**). Dark-gray bars represent the high likelihood stimulus (HLS) condition, while light gray bars represent the low likelihood stimulus condition (LLS). Error bars denote ±1 between-subjects *SEM*. (Color figure online)

Exact repetitions of stimulus identity across consecutive trials (labeled as stimulus priming above) were most likely to occur for high likelihood stimuli of the high likelihood cue type of each block, thus leading to potentially inflated evidence of learned attentional flexibility for high likelihood stimuli relative to low likelihood stimuli. Therefore, we next tested whether the subadditive relationship we observed in RTs was dependent on these exact repetition trials, running an identical 2 × 2 × 2 ANOVA to that described above, but excluding trials in which the cue stimulus was identical to that presented on trial *n-1*, and excluding the first trial of each block since it could not be a cue and/or stimulus repetition. As before, there were significant main effects of cue type, *F*(1,49) = 234.79, *p* < .001, η_p_^2^ = .827, stimulus identity likelihood, F(1,49) = 22.61, *p* < .001, η_p_^2^ = .316, and a significant interaction of cue type by block-wide shift likelihood, *F*(1,49) = 97.02, *p* < .001, η_p_^2^ = .664 (see Fig. 4A). Most interestingly, when removing the influence of exact cue stimulus repetitions, the three-way interaction failed to reach significance, *F*(1,49) = 1.16, *p* = .288, η_p_^2^ = .023, consistent with a serial relationship (see Fig. 4B). The remaining main effects and interactions also failed to reach significance (*F *values < 3.75, *p* values > .058).

As above, we conducted a parallel analysis with a Bayesian repeated-measures ANOVA. The best model again included parameters for cue type, block-wide shift likelihood, and stimulus identity likelihood, as well as the interaction of cue type by block-wide shift likelihood: BF_10_ = 5.050×10^30^ with respect to the null model (see Supplemental Table 2 for full model comparison). An analysis of effects again revealed decisive support for the interaction of cue type and block-wide shift likelihood, BF_inc_ = 2.771×10^10^, with moderate evidence against the interaction of block-wide shift likelihood and stimulus identity likelihood, BF_inc_ = 0.186 (see Table 3). However, when testing the three-way interaction, the evidence was inconclusive, BF_inc_ = 0.414.

**Table 3:** Analysis of effects excluding cue stimulus repetitions in Experiment 1

Effect	BF_inc_
CueType	2.523×10^17^
ShiftLike	0.622
StimLike	1,171.900
CueType × ShiftLike	2.771×10^10^
CueType × StimLike	0.927
ShiftLike × StimLike	0.186
CueType × ShiftLike × StimLike	0.414

*Note.* CueType denotes shift vs. hold, while ShiftLike and StimLike denote the shift and stimulus identity likelihoods, respectively. Models are only compared with equivalent models with higher-order interactions excluded

We next repeated the accuracy ANOVA with exact cue stimulus repetitions and the first trial of each block excluded. Participants were less accurate for shift attention trials than for hold attention trials, *F*(1,49) = 6.18, *p* = .016, η_p_^2^ = .112 (see Table 4). The three-way interaction failed to reach significance, *F*(1,49) = 3.17, *p* = .081, η_p_^2^ = .061, as did all other main effects and interactions (*F*s < 0.65, *p*s > .426).

**Table 4:** Behavioral accuracies excluding cue stimulus repetition trials in Experiment 1

	Low stimulus identity likelihood		High stimulus identity likelihood	
	Low shift likelihood	High shift likelihood	Low shift likelihood	High shift likelihood
Hold attention	92.18 (8.92)	92.62 (10.79)	92.81 (9.01)	91.11 (8.85)
Shift attention	90.08 (13.16)	89.83 (11.44)	90.14 (10.86)	90.71 (10.21)

*Note.* Mean percentage accuracies (*SD)*

**Fig. 5:**
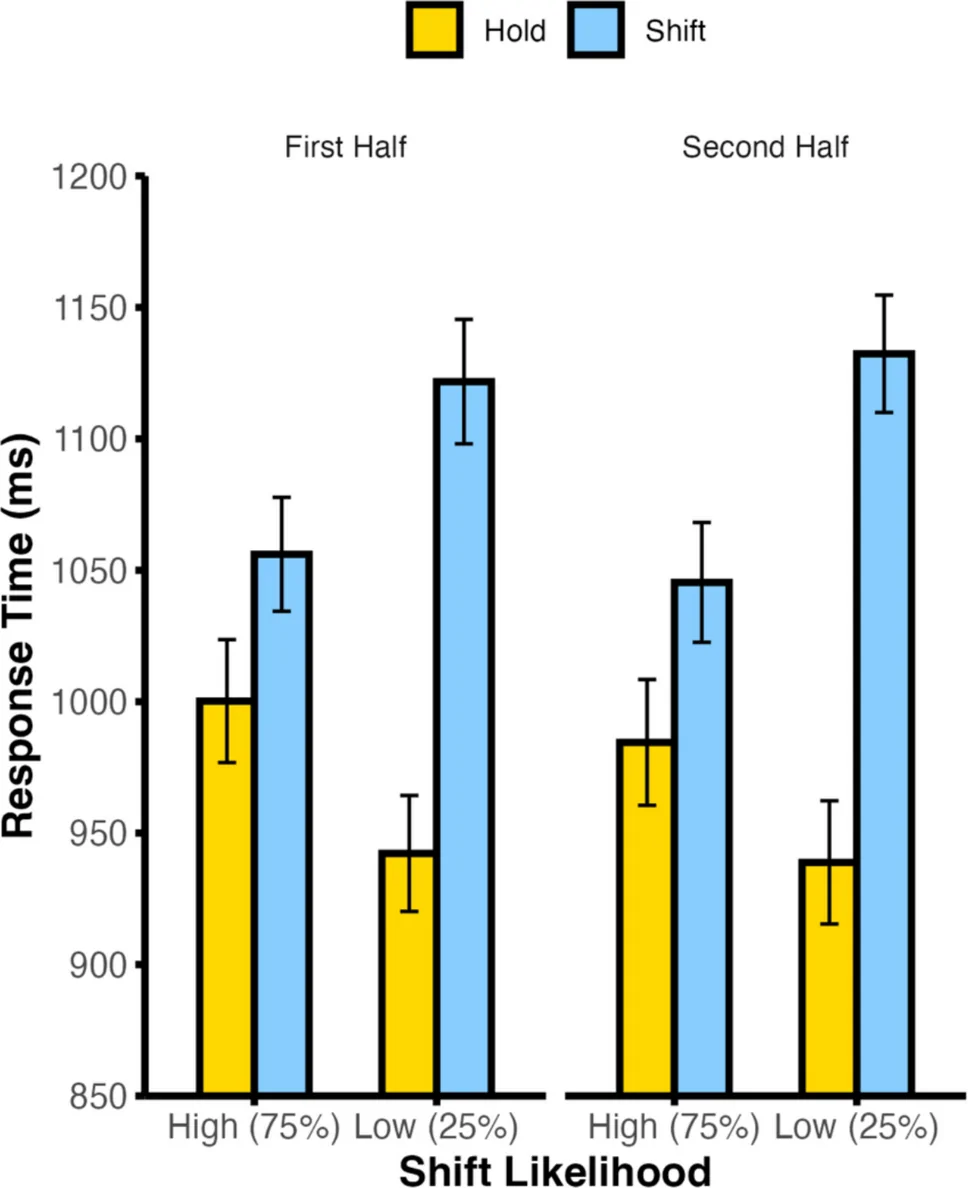
Behavioral response times as a function of cue type, block-wide shift likelihood, and block half in Experiment 1. Error bars denote ±1 between-subjects SEM

Lastly, we tested whether the modulation in shift costs that is attributable to learned shift readiness was stronger late in each block relative to the beginning of the block. This would suggest that shift readiness gradually builds throughout the block. While understanding the rate with which participants updated shift readiness predictions was not the focus of the current study, we divided each block into the first and second half of trials and tested whether the magnitude of shift costs differed between early and late trials within each block. Due to the small number of low stimulus identity likelihood trials per block, we focused this analysis on high probability stimuli only. As above, we excluded exact stimulus repetition trials and the first trial of each block for this analysis. We conducted a repeated-measures ANOVA, with the factors cue type (hold vs. shift), block-wide shift likelihood (high vs. low), and block half (first vs. second). As already noted, there was a significant main effect of cue type, *F*(1,49) = 189.77, *p*< .001, η_p_^2^ = .795, such that participants were slower on shift trials than on hold trials, and a significant interaction of cue type by shift likelihood, *F*(1,49) = 68.03, *p* < .001, η_p_^2^ = .581, such that the cost associated with shifting attention was smaller in high shift likelihood blocks than in low shift likelihood blocks (see Fig. 5). The three-way interaction failed to reach significance, *F*(1,49) = 0.19, *p* < .665, η_p_^2^ = .004, indicating that the shift cost difference between high and low shift likelihood blocks did not systematically differ between the first and second half of each block. No other main effects or interactions reached significance (*F* values < 1.71, *p* values > .197).

### Discussion

In Experiment 1, we tested the interaction of attention shifting readiness and stimulus identity prediction updating to determine whether these processes were constrained by a serial architecture, could proceed at least partially in parallel, or produced compounding behavioral costs. As expected, there were costs in both RT and accuracy associated with violations of attention shifting readiness as well as violations of stimulus identity expectations. Cue stimulus repetitions were associated with a significant speeding of RT that contributed to a subadditive interaction of shift readiness and stimulus identity updating processes when they were included in the analysis. Given our design, these trials disproportionately occurred for high likelihood stimuli of the high likelihood cue type and could therefore inflate the difference in shift costs between high and low shift likelihood blocks for high likelihood stimuli. When excluding exact cue stimulus repetitions, we observed no significant interaction of shift readiness and stimulus identity updating costs.

While the additive costs we observed when excluding exact cue stimulus repetitions were consistent with a serial processing framework, the Bayesian repeated-measures ANOVA did not provide interpretable evidence in favor of the null hypothesis. In Experiment 2, we addressed this concern in an extension of the task from Experiment 1 to increase our ability to find strong support either in favor of or opposed to the null hypothesis that shift likelihood and stimulus identity likelihood updating costs are additive.

## Experiment 2

In Experiment 2, we sought to follow up on the additive pattern we observed in Experiment 1 with a modified design to fully remove the influence of contiguous cue stimulus repetitions. To accomplish this goal, we introduced two additional shift attention and two additional hold attention cues, such that there was now a total of eight potential cues. As in Experiment 1, we again manipulated both shift likelihood across blocks and stimulus identity likelihood across participants. Unlike in Experiment 1, however, two shift cues served as high likelihood cues and two hold cues served has high likelihood cues for each participant. Furthermore, we increased our sample size as well as the total number of trials. As in the earlier study, evidence in favor of the null hypothesis that there is no three-way interaction of cue type, block-wide shift likelihood, and stimulus identity likelihood would support a serial processing account, while superadditive and subadditive interactions would support interference or parallel processing, respectively.

### Method

#### Participants

A total of 129 individuals (60 men, 69 women) ranging in age from 18 to 44 years (M = 32.8, SD = 6.25) completed the task online to the point of successfully submitting their data. A single individual completed Experiment 2 twice due to an error, and we only include data from their first session in all analyses reported here. Participants again received compensation of $12, and we used Prolific for participant recruitment with the same inclusion criteria as in Experiment 1. All procedures were again approved by the Wake Forest University Institutional Review Board, and participants provided consent with an electronic form. Given the increased difficulty of the task due to the additional cues, we had a higher percentage of participants with accuracy falling below the 70% cutoff we set in Experiment 1. A total of 34 participants were excluded for accuracies less than 70%, yielding a final sample of 95. Those participants excluded for low accuracy (*M* = 32.7, *SD* = 6.14) did not differ in age from those included in the final sample (*M* = 32.9, *SD* = 6.32), *t*(127) = 0.15, *p* = .882. None of the individuals included in Experiment 2 had participated in Experiment 1.

#### Stimuli

As in Experiment 1, we again controlled stimulus presentation with PsychoJs (Version 2023.1.3) running within the participant’s web browser and the task was hosted on Pavlovia. Participants again completed the credit card resizing task to account for differing monitor resolutions. The RSVP stimuli were identical to those used in Experiment 1 except for the cue stimuli. The letters *A*, *B*, *C*, *D*, *R*, *S*, *T*, and *U* served as shift and hold cues, with the constraint that the first four letters of the alphabet (*A*–*D*) were always either all shift cues or all hold cues, and the remaining letters (*R*–*U*) signaled the opposite operation. Rather than creating a unique mnemonic for each of the four shift and four hold cues—many of which would involve unrelated letters—we opted to group the cues into two alphabetical clusters. This approach supported ease of learning by allowing participants to associate each cluster with a single operation and avoided overlap with the response keys (“z” and “m”) used for parity judgments. This change from cues with mnemonic attachment in Experiment 1 (“S”–Shift, “C”–Change) to random assignment in Experiment 2 increased the overall difficulty of the task, but extends the findings of Experiment 1 to a scenario where there are no preexisting associations between stimulus and cue meaning. Each frame of the RSVP task was again presented for 250 ms, with no gap between frames.

#### Procedure

All aspects of the procedure were identical to Experiment 1, except where noted below. The distractor interval was randomly selected on each trial to either be 1,000 ms or 2,000 ms. Participants were divided into groups according to

- Which stimuli had a high or low likelihood of appearing,
- Whether they began with a low or high shift likelihood block first (subsequently, the task alternated between low and high likelihood across consecutive blocks as in Experiment 1), and
- Whether *A*–*D* cued shifts or holds, with *R*–*U* cuing the opposite operation.

To simplify the number of possible combinations, we restricted the stimulus probabilities such that *A* and *B* always had the same stimulus identity likelihood classification. Likewise, we also equated the likelihood of *C* and *D*, as well as *R* and *S* and *T* and *U*. A randomization error caused one of the 16 potential groups to be assigned 0 participants and another to be assigned 16 participants in the final sample, with the remaining 14 groups having between 4 to 9 participants each (*M* = 5.94, *SD* = 3.32 across all potential groups).

Lastly, we slightly increased the number of trials relative to Experiment 1 so that participants completed eight blocks of 64 trials each. Within each block participants received 75% shift cues or 25% shift cues. Two shift stimuli and two hold stimuli were each presented on 37.5% of shift and hold trials, respectively, with the remaining stimuli each presented on 12.5% of trials. Importantly, we controlled stimulus priming by assigning cues such that there were never any exact stimulus repetitions across consecutive trials. This procedure ensured that all trials contributed to the main analyses of interest. Participants completed a scaffolded practice as in Experiment 1. Unlike the main task, the distractor interval could be 1,000 ms, 1,500 ms, or 2,000 ms during this practice.

#### Data Analysis

We again primarily focused on an analysis of RTs to determine whether there were additive, subadditive, or superadditive costs associated with shift likelihood and stimulus identity likelihood violations. Only RTs from trials with a correct response were included in the analysis and we again trimmed all RTs less than 200 ms, resulting in a reduction of less than 1% of all trials with an accurate response.

### Results

**Fig. 6:**
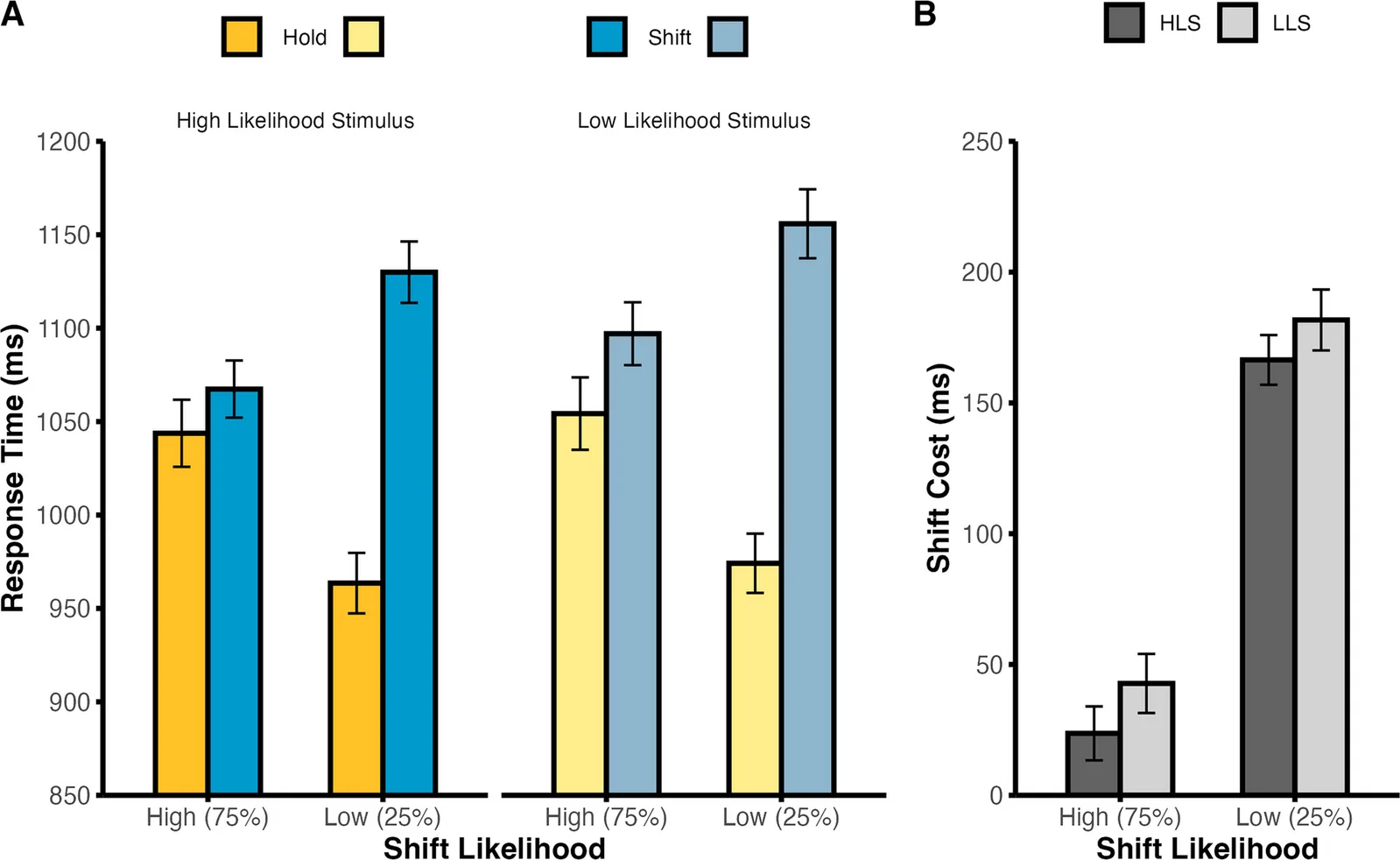
Behavioral response time analysis in Experiment 2. Behavioral response times for trials with a high likelihood stimulus (**A**; left) versus trials with a low likelihood stimulus (**A**; right). Comparison of the shift costs between high and low shift likelihood blocks for each stimulus identity likelihood condition (**B**). Dark-gray bars represent the high likelihood stimulus (HLS) condition, while light-gray bars represent the low likelihood stimulus condition (LLS). Error bars denote ±1 between-subjects *SEM*. (Color figure online)

As in Experiment 1, we first analyzed RTs with a 2 × 2 × 2 repeated-measures ANOVA, with factors cue type (hold vs. shift), block-wide shift likelihood (high vs. low), and stimulus identity likelihood (high vs. low). There were again significant main effects of cue type, *F*(1,94) = 161.87, *p* < .001, η_p_^2^ = .633, and stimulus identity likelihood, *F*(1,94) = 17.57, *p* < .001, η_p_^2^ = .157, such that participants were slower on shift trials than on hold trials and slower on low stimulus identity likelihood trials than on high stimulus identity likelihood trials. The main effect of shift likelihood approached significance, *F*(1,94) = 3.33, *p* = .071, η_p_^2^ = .034. Shift costs again varied according to the shift likelihood, as indicated by a significant interaction of cue type by shift likelihood, *F*(1,94) = 216.23, *p* < .001, η_p_^2^ = .697. There was also a significant interaction of cue type by stimulus identity likelihood, *F*(1,94) = 5.78, *p* = .018, η_p_^2^ = .058, but no significant interaction of shift likelihood by stimulus identity likelihood, *F*(1,94) = 0.08, *p* = .782, η_p_^2^ < .001. Most interestingly, the three-way interaction failed to reach statistical significance, *F*(1,94) = 0.07, *p* = .791, η_p_^2^ < .001 (see Fig. 6).

To better account for the evidence in favor of or against the three-way interaction, we next subjected the RT data to a Bayesian repeated-measures ANOVA. The model that best accounted for the data included regressors for cue type, shift likelihood, stimulus identity likelihood, the interaction of cue type by shift likelihood, and the interaction of cue type by stimulus identity likelihood: BF_10_ = 3.066×10^44^ with respect to the null model (see Supplemental Table 3 for the full model comparison). We then conducted an analysis of effects as in Experiment 1. There was decisive support for the interaction of cue type by block-wide shift likelihood, BF_inc_ = 1.150×10^23^, moderate evidence against the interaction of block-wide shift likelihood by stimulus identity likelihood, BF_inc_ = 0.130, and inconclusive evidence regarding the interaction of cue type by stimulus identity likelihood, BF_inc_ = 1.437. In extension of the results from Experiment 1, we found moderate evidence in favor of the null hypothesis that there was no three-way interaction of cue type, shift likelihood, and stimulus identity likelihood, BF_inc_ = 0.172 (see Table 5 for the full analysis of effects).

**Table 5:** Analysis of effects in Experiment 2

Effect	BF_inc_
CueType	1.179×10^19^
ShiftLike	0.638
StimLike	242.579
CueType × ShiftLike	1.150×10^23^
CueType × StimLike	1.437
ShiftLike × StimLike	0.130
CueType × ShiftLike × StimLike	0.172

*Note.* CueType denotes shift vs. hold, while ShiftLike and StimLike denote the shift and stimulus identity likelihoods, respectively. Models are only compared with equivalent models with higher-order interactions excluded.

We next analyzed behavioral accuracies with a repeated-measures ANOVA that was identical to the analysis of RTs. Participants were less accurate for trials with low probability stimuli than for trials with high probability stimuli, *F*(1,94) = 7.09, *p* = .009, η_p_^2^ = .070 (see Table 6). The main effect of cue type approached significance, *F*(1,94) = 2.85, p = .095, η_p_^2^ = .029. There was also an interaction of cue type by shift likelihood that mirrored the pattern of results found with RTs, *F*(1,94) = 28.56, *p* < .001, η_p_^2^ = .233. No other main effects or interactions reached significance (*F* values < 1.03, *p* values > .313).

**Table 6:** Behavioral accuracies in Experiment 2

	Low stimulus identity likelihood		High stimulus identity likelihood	
	Low shift likelihood	High shift likelihood	Low shift likelihood	High shift likelihood
Hold attention	90.68 (9.51)	87.17 (12.07)	91.17 (8.14)	88.75 (9.69)
Shift attention	86.38 (13.30)	88.53 (11.98)	87.50 (10.57)	89.31 (11.39)

*Note. *Mean percentage accuracies (*SD*).

**Fig. 7:**
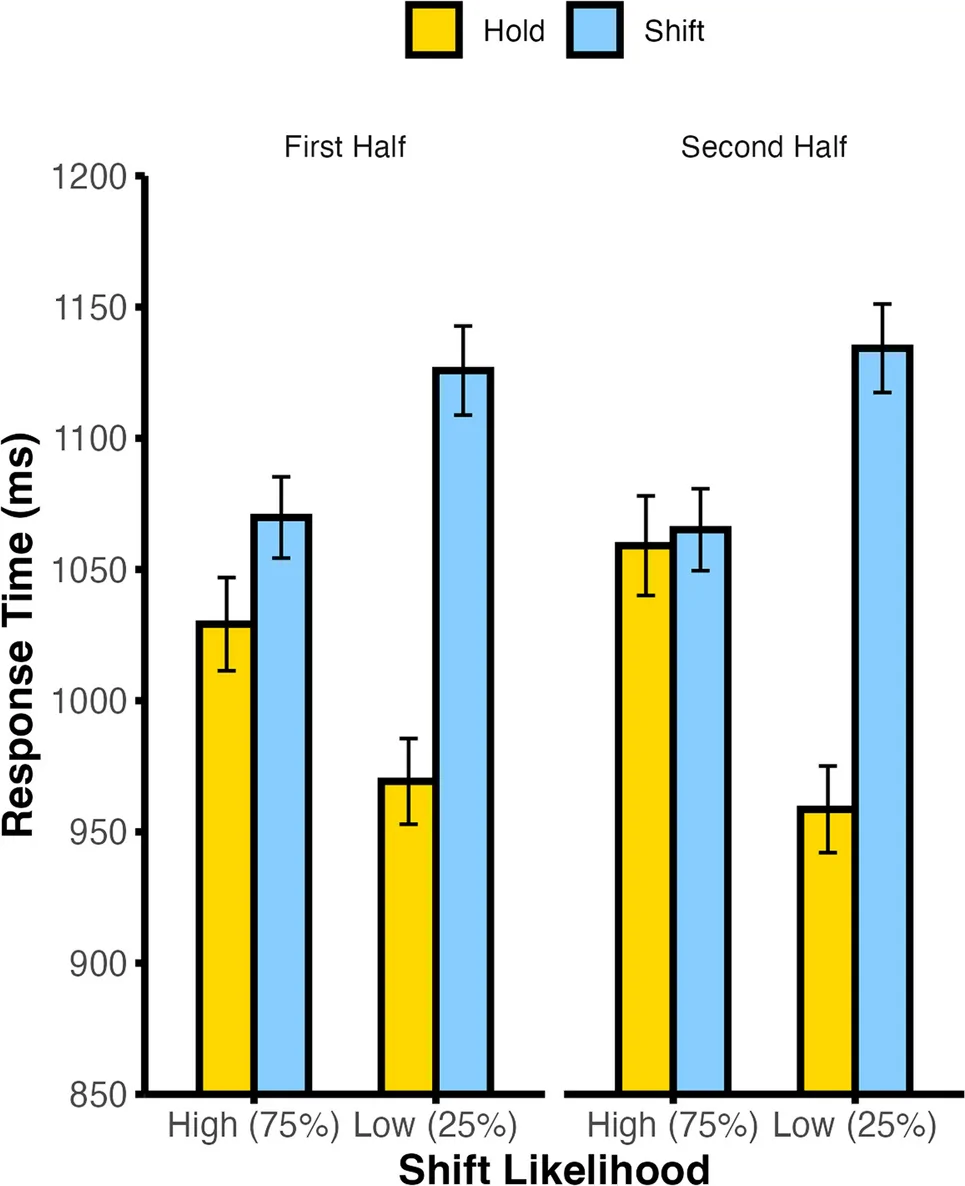
Behavioral response times as a function of cue type, block-wide shift likelihood, and block half in Experiment 2. Error bars denote ±1 between-subjects *SEM*. (Color figure online)

We again tested whether the shift cost difference between high and low shift likelihood blocks varied across the first and second half of each block with a repeated-measures ANOVA for high stimulus identity likelihood trials only as in Experiment 1. As in the previous analysis, there was a significant main effect of cue type, *F*(1,94) = 122.47, *p* < .001, η_p_^2^ = .566, and an interaction of cue type by shift likelihood, *F*(1,94) = 214.52, *p* < .001, η_p_^2^ = .695. A significant interaction of shift likelihood by block half, *F*(1,94) = 4.39, *p* = .039, η_p_^2^ = .045, revealed that there was a greater reduction in overall RT for low shift likelihood blocks relative to high shift likelihood blocks in the second half of each block than in the first. Most interestingly, the three-way interaction was significant, *F*(1,94) = 15.06, *p* < .001, η_p_^2^ = .138. Adjustments in shift readiness solidified across the block such that there was a larger block-wide shift likelihood difference when comparing second halves than when comparing first halves (see Fig. 7). The remaining main effect and interaction did not reach significance (*F* values < 2.70, *p* values > .104). A follow-up ANOVA for first half of block trials only revealed that while the magnitude of learned flexibility adjustments increased over the course of the block, there was a still a significant modulation of shift readiness in the first half of the block alone, *F*(1,94) = 117.78, *p* < .001, η_p_^2^ =.556.

### Discussion

In Experiment 2, we introduced two additional cues of each type, yielding four shift and four hold cues, so that we could remove the possibility of exact cue repetitions across consecutive trials. With these exact repetitions removed from the experimental design we again found no significant three-way interaction between cue type, block-wide shift likelihood, and stimulus identity likelihood. The inclusion of additional cues required us to choose a stimulus set that did not have strong mnemonic associations, as had the stimuli in Experiment 1. This change likely contributed to the larger number of participants falling below the 70% overall accuracy inclusion cutoff. Nonetheless, the pattern of RT results suggests that additive costs associated with shift likelihood and stimulus identity prediction errors are not dependent on preexisting stimulus–response associations. Furthermore, a Bayesian repeated-measures ANOVA revealed moderate support for the null hypothesis that no three-way interaction exists over the alternative. Unlike Experiment 1, we observed evidence that the difference in shift costs between high and low shift likelihood blocks was larger for trials coming in the second half of the block than the first. This pattern suggests that participants continued adjusting their shift readiness throughout the course of the block, with stronger readiness predictions forming as more trials in the block were completed. However, shift costs were still significantly smaller for high shift likelihood blocks than for low shift likelihood blocks in the first half of the block, suggesting that individuals began updating shift predictions early after each probability change.

## General discussion

In the current study, we tested the interaction of shift readiness and stimulus identity prediction errors to determine whether the costs associated with these unexpected outcomes were additive or significantly interacted (see Kessler, 2017; Sali & Egner, 2020; da Silva Souza et al., 2012 for similar designs). To do this, we adopted a design with multiple shift and hold cues, as is common in the task-switching literature (Forstmann et al., 2007; Logan & Bundesen, 2003; Mayr, 2006; Mayr & Kliegl, 2003; Schneider & Logan, 2007). Experiment 1 contained two shift and two hold cues (one high probability and one low probability for shift and hold each), and this design led to some consecutive trials having the same cue stimulus. When including these exact repetition trials, we observed robust evidence of a subadditive relationship between block-wide shift likelihood and stimulus identity likelihood violations. However, when excluding these exact repetition trials, there was no significant interaction. In Experiment 2, we broadened the cue space to include four cues for each type (two high probability shift/hold cues and two low probability shift/hold cues) allowing us to constrain the trial setup so that exact cue stimulus repetitions never occurred. Under these conditions, we observed support for the null hypothesis—that shift likelihood updating costs and stimulus identity updating costs were additive—that was approximately six times more likely than the alternative that an interaction existed.

The additive costs observed here are consistent with multiple potential serial processing architectures. First, the output of one updating operation may be needed as an input to the other, creating a serial dependence. Although our paradigm is not equipped to answer which violation detection process must be completed before the other, one possibility is that individuals first update stimulus identity predictions during an initial classification of the cue stimulus and that this process is later used to update shift readiness predictions. In this example, the updating mechanisms for shift likelihood and stimulus identity must be carried out in a particular order. Alternatively, it is possible that the two updating operations could be carried out in any order but compete for a shared processing bottleneck. Under this interpretation, the bottleneck must prevent any component processes of the second-initiated updating operation from beginning prior to completion of the first update, since any component processes that occur in parallel could lead to a reduction in the total completion time (Pashler, 1994). For example, there may exist a common updating mechanism that can work on only one set of predictions at a time, requiring the other to wait in a queue until the bottleneck is free. Lastly, while the serial architectures described here are most parsimonious, we cannot completely rule out the possibility that some component processes are executed in parallel, but that this time savings is offset by an increase in time needed to shift a shared mechanism from one operation to the other. However, this interpretation is unlikely since the time savings of parallel processing would need to equal the cost associated with shifting the shared mechanism.

The current data suggest that predictions of stimulus identity and shift readiness are likely coded as distinct representations. If this were not the case, we would expect participants to demonstrate no additional slowing for stimuli that were both unlikely and cued an unlikely attention operation (shifting vs. holding) relative to those that were simply unlikely stimuli. While some studies have used consistent stimulus-attention operation mappings, such as the letter “S” always cuing an attention shift, others have used designs where the stimulus cues a location instead of the operation itself (Sali et al., 2022). For example, the letter “L” could be used to cue the left location, indicating a shift if the participant is currently attending to the right or a hold if the participant is already attending to the left. Individuals still demonstrate learned shift readiness adjustments in tasks without fixed stimulus–operation mappings, suggesting that an expectation to shift attention cannot be purely explained by an expectation for a particular cue stimulus. However, it is possible that cue stimulus identity expectations are re-coded dynamically based on the currently attended location. The current results do not support this interpretation. Instead, the additive costs identified here suggest that identity and shift readiness expectations are processed individually with a serial architecture such that one process must end before the other begins. We cannot rule out the possibility that shift readiness representations are at least partially linked to stimulus representations. For example, stimulus identity predictions may update before being carried forward to downstream processes that integrate stimulus identities with shift readiness. This question remains outside the scope of the current study and will be an important topic for future research.

While our study is the first to our knowledge to manipulate spatial attention shifting and stimulus identity likelihoods simultaneously, our findings parallel those from the task-switching literature. Manipulations of switch likelihood have revealed that switch costs increase as the likelihood of switching decreases (e.g., Braem & Egner, 2018; Crump & Logan, 2010; Dreisbach & Haider, 2006), and that the switch likelihood can determine the extent to which participants’ performance is best explained by cue priming alone, or reflects a combination of cue encoding and task set switching processes (Forstmann et al., 2007; Mayr, 2006; Monsell & Mizon, 2006). Furthermore, the subadditive relationship that we observed in Experiment 1 when including cue stimulus repetition trials is consistent with cue switch costs (Logan & Bundesen, 2003; Schneider & Logan, 2007). Our design extends these findings to the domain of spatial attentional control and adds a manipulation of cue likelihood to better understand how individuals behave when faced with multiple prediction errors.

Our inferences about the additivity of costs associated with shift and stimulus identity likelihood violations relied on testing the factorial interaction (see Townsend, 1990), which is an extension of Sternberg’s additive factor method (Sternberg, 1969). While the Bayesian evidence in favor of an additive relationship can be interpreted as support for serial processing, more sophisticated analytical techniques, which would require a modified experimental design, are needed to discern other potential processing architectures more completely. For example, systems factorial technology (SFT) builds on the interaction contrast we have conducted here by using survivor functions of RT distributions to compute a survivor interaction contrast (SIC; Harding et al., 2016; Little et al., 2017). To estimate RT distributions, this procedure would require several orders of magnitude more trials in the rarest conditions than in the current study, which had 15 or 16 total trials per participant before excluding trials with errors in Experiments 1 and 2, respectively. For example, while a serial self-terminating architecture in which only one of two queued operations needs to finish for the overall process to complete yields an SIC that is constantly zero, a serial exhaustive architecture in which sequential subprocesses must finish before the overall process is complete produces an SIC that has negative deflection followed by a positive deflection (Townsend & Nozawa, 1995). Despite our inability to conduct this analysis, in the current study, participants needed to both detect the cue and apply the corresponding attention operation (shift vs. hold) before being able to make their response, making a serial self-terminating architecture unlikely. SFT could also better characterize the subadditive relationship we observed in Experiment 1 when including cue stimulus repetitions across trials as parallel exhaustive (the overall process is finished when the longest sub-process is complete) or coactive (the subprocesses share subtasks) architectures (Harding et al., 2016).

In the current study, we manipulated shift readiness by varying the likelihood of shift cues across blocks of trials. Conversely, stimulus identity varied across participants. Additional research will be needed to determine whether the additive relationship we observed applies to situations in which the statistical regularities for both domains are varied at the same temporal timescale. For example, it is possible that by holding stimulus identity likelihoods constant across the experiment that the identity updating mechanism was shielded from interference resulting from the shorter-term shift readiness learning. One possible solution would be to compare the processing architecture observed here to cases in which stimulus identity also varies across blocks or in which shift readiness varies with consistent probabilities across the experiment. For example, shift readiness is known to vary based on temporal cue expectation learning (see Sali et al., 2015) and this form of contextual learning would allow shift readiness to vary in a consistent fashion through the experimental session.

The subadditive pattern we observed when including cue stimulus repetitions is consistent with evidence that attention is automatically drawn to stimuli with repeating features in spatial visual search tasks (Maljkovic & Nakayama, 1994). Likewise, individuals experience temporal position priming in RSVP tasks such that performance is facilitated when the temporal position of a target in a continuous stream is repeated (Yashar & Lamy, 2013). Given these other examples of intertrial effects speeding performance, it is possible that repeating cue stimulus features may speed either, or both, stimulus identity or shift readiness prediction updating, or allow the two processes to occur at least partially in parallel. Intertrial priming effects are known to span multiple trials (Allenmark et al., 2021) and our analysis approach in Experiment 1 intentionally only excluded the second of consecutive trials with an exact stimulus repetition. Our reasoning for this decision was that our manipulation of shift readiness and stimulus identity probabilities necessitates longer-scale priming effects. In fact, computational modeling of RL often requires fitting a parameter, referred to as the learning rate, that accounts for individual differences in the tendency to weight immediate vs. longer time scale evidence when updating predictions. Likewise, although our manipulation in Experiment 2 removed the possibility of stimulus identity repetitions across consecutive trials, it was still more likely that an individual would have experienced a repetition between high likelihood cues in recent trial history and these sequence effects have been linked to modulations of switch costs in the task-switching literature (Schneider & Logan, 2007). It remains an open question whether individuals who tend to weigh longer-scale trial histories more heavily than immediate history when updating shift predictions do the same when updating stimulus identity predictions.

The predictive processing framework stipulates that individuals generate models of the world that guide perception with the goal of minimizing prediction error (see Ficco et al., 2021, for a review). Under this umbrella, active inference theories suggest that individuals seek to explain the most likely cause of moment-by-moment perceptions by minimizing expected free energy, the upper bound in the level of surprise that an observer may experience for a given perception in response to the observer’s actions (Clark, 2013; den Ouden et al., 2010; Friston, 2010). In other words, individuals choose to behave in ways that maximize the expected reward they will receive or information they will gain, given their model of the world, and this model is updated as new sensory information is acquired (Friston et al., 2017). While our current results are consistent with this framework, the ubiquity of predictive processing theories for broadly explaining human behavior is not without criticism (Sun & Firestone, 2020). Here, we suggest a narrower interpretation: Previously encountered stimulus features and shift likelihoods influence moment-by-moment performance, likely through a prediction-updating mechanism, with additive costs associated with unexpected cues to shift/hold attention and unexpected stimulus identities.

While our results are consistent with the predictive processing framework, we cannot disentangle from our data alone whether the additive costs we observed are related to (a) detection of a surprising shift/stimulus outcome, (b) overcoming the violation of expectations, and/or (c) updating future predictions. The total cost in RT associated with unexpected stimulus identities and unexpected cue types likely reflects a combination of these processes. For example, unexpected stimuli are afforded increased attentional priority and are known to automatically capture attention (Corbetta et al., 2008; Corbetta & Shulman, 2002; Theeuwes, 1992; Yantis & Jonides, 1984). Attention may automatically select unexpected stimuli, potentially contributing to previous findings implicating stimulus-driven attentional orienting mechanisms to violations of shifting expectations (Sali et al., 2020, 2024). One possibility is therefore that this automatic selection of an unexpected stimulus pauses processing, and that a disengagement from stimulus processing is needed before shift readiness updating can occur. Alternatively, it is possible that the costs we observed here relate more strongly to the process of overriding the preparatory responses that are planned given a pretrial expectation of the upcoming stimulus identity and cue type. Finally, RL models effectively explain trial-by-trial variability in shift readiness and stimulus expectations and it is possible that the costs we observed are most directly related to the use of prediction errors to better inform future predictions. While these three explanations are not mutually exclusive, future research using computational modeling or neuroimaging may offer insights into the relative contributions.

In Experiment 2, we observed evidence that the difference in shift cost attributable to block-wide shift likelihood was stronger in the second half of each block relative to the first. The success of RL models in accounting for trial-by-trial shift readiness in spatial orienting (Sali et al., 2020) and task-switching (Sali et al., 2024) paradigms suggests that individuals regularly update predictions about upcoming demands. The block half by shift likelihood by cue type interaction likely reflects this ongoing process of adjusting shift readiness based on trial history. Given our alternating probabilities design, with each new block, participants needed to adjust their shift expectations. We did not design the current study to test the length of time needed to adjust shifting expectations. However, there was no three-way interaction in Experiment 1, suggesting participants demonstrated equivalent modulations of shift readiness across block halves, and shift costs varied according to the block-wide shift likelihood even when restricting the analysis to the first half of each block when a significant interaction was present in Experiment 2. These findings suggest that individuals adjusted to the new probability structure rapidly. Future research could apply RL models to understand trial-by-trial shift predictions and stimulus identity predictions as a function of trial history to better capture performance when the strength of shift predictions (beginning of a block) or stimulus identity predictions (beginning of the experiment) are relatively weak. Importantly, the strengthening of shift predictions over the course of a block cannot account for our current findings since low likelihood stimuli were equally likely to appear in the first and second half of each block.

In the current study, we used an interaction model to test whether two forms of prediction updating—namely, spatial attention shifting readiness and stimulus identity, are constrained by a serial architecture, proceed in parallel, or interfere with one another. Using a design in which shift likelihood varied across blocks and stimulus identity likelihood was held constant, we found evidence of serial processing when excluding trials with exact cue stimulus repetitions. Our results hold implications for understanding how different environmental regularities are integrated to shape adaptive behavior.

## Supplementary Information

Below is the link to the electronic supplementary material.

Supplementary file1 (DOCX 22.9 KB)

## Data Availability

The data and materials for this experiment are available online (https://osf.io/zdtvc/).

## Footnotes

1 In both Experiments 1 and 2, participants were able to restart the task by either returning to the online consent form or refreshing the browser window running the task. In some cases, the participant was randomly assigned to a different stimulus probability condition upon restarting. We have elected to retain these participants since any restarts likely occurred early in the task and would create noise in the data that would only weaken stimulus likelihood learning (Experiments 1 and 2) and cue type recognition (Experiment 2). Here, and in Experiment 2, we do not report individuals who ended the study prior to submitting their data or experienced a technical error.

## Code availability

All stimulus presentation and analysis code are available online (https://osf.io/zdtvc/).

## References

[CR1] Allenmark, F., Gokce, A., Geyer, T., Zinchenko, A., Müller, H. J., & Shi, Z. (2021). Inter-trial effects in priming of pop-out: Comparison of computational updating models. *PLOS Computational Biology,**17*(9), e1009332. 10.1371/journal.pcbi.1009332 34478446 10.1371/journal.pcbi.1009332 PMC8445473

[CR2] Bar, M. (2007). The proactive brain: Using analogies and associations to generate predictions. *Trends in Cognitive Sciences,**11*(7), 280–289. 10.1016/j.tics.2007.05.005 17548232 10.1016/j.tics.2007.05.005

[CR3] Bledowski, C., Prvulovic, D., Hoechstetter, K., Scherg, M., Wibral, M., Goebel, R., & Linden, D. E. J. (2004). Localizing P300 generators in visual target and distractor processing: A combined event-related potential and functional magnetic resonance imaging study. *The Journal of Neuroscience,**24*(42), 9353–9360. 10.1523/JNEUROSCI.1897-04.2004 15496671 10.1523/JNEUROSCI.1897-04.2004 PMC6730097

[CR4] Braem, S., & Egner, T. (2018). Getting a grip on cognitive flexibility. *Current Directions in Psychological Science,**27*(6), 470–476. 10.1177/0963721418787475 30555214 10.1177/0963721418787475 PMC6291219

[CR5] Chiu, Y. C., & Egner, T. (2017). Cueing cognitive flexibility: Item-specific learning of switch readiness. *Journal of Experimental Psychology: Human Perception and Performance,**43*(12), 1950–1960. 10.1037/xhp0000420 28406686 10.1037/xhp0000420 PMC5640457

[CR6] Chiu, Y. C., Jiang, J., & Egner, T. (2017). The caudate nucleus mediates learning of stimulus-control state associations. *Journal of Neuroscience,**37*, 1028–1038. 10.1523/jneurosci.0778-16.2016 28123033 10.1523/JNEUROSCI.0778-16.2016 PMC5296776

[CR7] Clark, A. (2013). Whatever next? Predictive brains, situated agents, and the future of cognitive science. *The Behavioral and Brain Sciences,**36*(3), 181–204. 10.1017/S0140525X12000477 23663408 10.1017/S0140525X12000477

[CR8] Corbetta, M., & Shulman, G. L. (2002). Control of goal-directed and stimulus-driven attention in the brain. *Nature Reviews Neuroscience,**3*, 201–215. 10.1038/nrn755 11994752 10.1038/nrn755

[CR9] Corbetta, M., Patel, G., & Shulman, G. L. (2008). The reorienting system of the human brain: From environment to theory of mind. *Neuron,**58*, 306–324. 10.1016/j.neuron.2008.04.017 18466742 10.1016/j.neuron.2008.04.017 PMC2441869

[CR10] Crump, M. J. C., & Logan, G. D. (2010). Contextual control over task-set retrieval. *Attention, Perception, & Psychophysics,**72*, 2047–2053. 10.3758/bf03196681 10.3758/bf03196681 21097849

[CR11] da Silva Souza, A., Oberauer, K., Gade, M., & Druey, M. D. (2012). Processing of representations in declarative and procedural working memory. *Quarterly Journal of Experimental Psychology (2006),**65*(5), 1006–1033. 10.1080/17470218.2011.640403 22332900 10.1080/17470218.2011.640403

[CR12] den Ouden, H. E. M., Daunizeau, J., Roiser, J., Friston, K. J., & Stephan, K. E. (2010). Striatal prediction error modulates cortical coupling. *Journal of Neuroscience,**30*(9), 3210–3219. 10.1523/JNEUROSCI.4458-09.2010 20203180 10.1523/JNEUROSCI.4458-09.2010 PMC3044875

[CR13] Downar, J., Crawley, A. P., Mikulis, D. J., & Davis, K. D. (2000). A multimodal cortical network for the detection of changes in the sensory environment. *Nature Neuroscience,**3*(3), 277–283. 10.1038/72991 10700261 10.1038/72991

[CR14] Dreisbach, G., & Haider, H. (2006). Preparatory adjustment of cognitive control in the task switching paradigm. *Psychonomic Bulletin & Review,**13*(2), 334–338. 10.3758/bf03193853 16893004 10.3758/bf03193853

[CR15] Ficco, L., Mancuso, L., Manuello, J., Teneggi, A., Liloia, D., Duca, S., & Cauda, F. (2021). Disentangling predictive processing in the brain: A meta-analytic study in favour of a predictive network. *Scientific Reports,**11*(1), 16258. 10.1038/s41598-021-95603-5 34376727 10.1038/s41598-021-95603-5 PMC8355157

[CR16] Forstmann, B. U., Brass, M., & Koch, I. (2007). Methodological and empirical issues when dissociating cue-related from task-related processes in the explicit task-cuing procedure. *Psychological Research,**71*(4), 393–400. 10.1007/s00426-005-0040-4 16397813 10.1007/s00426-005-0040-4

[CR17] Friston, K. (2010). The free-energy principle: A unified brain theory? *Nature Reviews Neuroscience,**11*(2), 127–138. 10.1038/nrn2787 20068583 10.1038/nrn2787

[CR18] Friston, K., FitzGerald, T., Rigoli, F., Schwartenbeck, P., & Pezzulo, G. (2017). Active inference: A process theory. *Neural Computation,**29*(1), 1–49. 10.1162/NECO_a_00912 27870614 10.1162/NECO_a_00912

[CR19] Harding, B., Goulet, M.-A., Jolin, S., Tremblay, C., Villeneuve, S.-P., & Durand, G. (2016). Systems factorial technology explained to humans. *The Quantitative Methods for Psychology,**12*(1), 39–56. 10.20982/tqmp.12.1.p039

[CR20] Kessler, Y. (2017). The role of working memory gating in task switching: A procedural version of the reference-back paradigm. *Frontiers in Psychology*, *8*. 10.3389/fpsyg.2017.02260 10.3389/fpsyg.2017.02260 PMC5742995 29312095

[CR21] Lakens, D., & Caldwell, A. R. (2021). Simulation-based power analysis for factorial analysis of variance designs. *Advances in Methods and Practices in Psychological Science,**4*(1), 251524592095150. 10.1177/2515245920951503

[CR22] Leboe, J. P., Wong, J., Crump, M., & Stobbe, K. (2008). Probe-specific proportion task repetition effects on switching costs. *Perception & Psychophysics,**70*, 935–945. 10.3758/pp.70.6.935 18717381 10.3758/pp.70.6.935

[CR23] Little, D. R., Altieri, N., Fifić, M., & Yang, Z. (Eds.). (2017). *Systems factorial technology: A theory driven methodology for the identification of perceptual and cognitive mechanisms*. Academic.

[CR24] Logan, G. D., & Bundesen, C. (2003). Clever homunculus: Is there an endogenous act of control in the explicit task-cuing procedure? *Journal of Experimental Psychology: Human Perception and Performance,**29*(3), 575–599. 10.1037/0096-1523.29.3.575 12848327 10.1037/0096-1523.29.3.575

[CR25] Maljkovic, V., & Nakayama, K. (1994). Priming of pop-out: I. *Role of features. Memory & Cognition,**22*(6), 657–672. 10.3758/BF03209251 7808275 10.3758/bf03209251

[CR26] Marois, R., Leung, H. C., & Gore, J. C. (2000). A stimulus-driven approach to object identity and location processing in the human brain. *Neuron,**25*(3), 717–728. 10.1016/s0896-6273(00)81073-9 10774738 10.1016/s0896-6273(00)81073-9

[CR27] Mayr, U. (2006). What matters in the cued task-switching paradigm: Tasks or cues? *Psychonomic Bulletin & Review,**13*(5), 794–799. 10.3758/BF03193999 17328375 10.3758/bf03193999

[CR28] Mayr, U., & Kliegl, R. (2003). Differential effects of cue changes and task changes on task-set selection costs. *Journal of Experimental Psychology: Learning, Memory, and Cognition,**29*(3), 362–372. 10.1037/0278-7393.29.3.362 12776747 10.1037/0278-7393.29.3.362

[CR29] Miller, J. (2023). Outlier exclusion procedures for reaction time analysis: The cures are generally worse than the disease. *Journal of Experimental Psychology: General,**152*(11), 3189–3217. 10.1037/xge0001450 37498697 10.1037/xge0001450

[CR30] Monsell, S., & Mizon, G. A. (2006). Can the task-cuing paradigm measure an endogenous task-set reconfiguration process? *Journal of Experimental Psychology: Human Perception and Performance,**32*(3), 493–516. 10.1037/0096-1523.32.3.493 16822121 10.1037/0096-1523.32.3.493

[CR31] Pashler, H. (1994). Dual-task interference in simple tasks: Data and theory. *Psychological Bulletin,**116*(2), 220–244. 10.1037/0033-2909.116.2.220 7972591 10.1037/0033-2909.116.2.220

[CR32] Peirce, J., Gray, J. R., Simpson, S., MacAskill, M., Höchenberger, R., Sogo, H., ..., & Lindeløv, J. K. (2019). PsychoPy2: Experiments in behavior made easy. *Behavior Research Methods*, *51*(1), 195–203. 10.3758/s13428-018-01193-y 10.3758/s13428-018-01193-y PMC6420413 30734206

[CR33] Rao, R. P. N., & Ballard, D. H. (1999). Predictive coding in the visual cortex: A functional interpretation of some extra-classical receptive-field effects. *Nature Neuroscience,**2*(1), 79–87. 10.1038/4580 10195184 10.1038/4580

[CR34] Sali, A. W., & Egner, T. (2020). Declarative and procedural working memory updating processes are mutually facilitative. *Attention, Perception & Psychophysics,**82*(4), 1858–1871. 10.3758/s13414-019-01887-1 10.3758/s13414-019-01887-1 PMC7302969 31875313

[CR35] Sali, A. W., Anderson, B. A., & Yantis, S. (2015). Learned states of preparatory attentional control. *Journal of Experimental Psychology: Learning, Memory, and Cognition,**41*, 1790–1805. 10.1037/xlm0000146 26076326 10.1037/xlm0000146 PMC4630134

[CR36] Sali, A. W., Courtney, S. M., & Yantis, S. (2016). Spontaneous fluctuations in the flexible control of covert attention. *Journal of Neuroscience,**36*, 445–454. 10.1523/jneurosci.2323-15.2016 26758836 10.1523/JNEUROSCI.2323-15.2016 PMC4710768

[CR37] Sali, A. W., Jiang, J., & Egner, T. (2020). Neural mechanisms of strategic adaptation in attentional flexibility. *Journal of Cognitive Neuroscience,**32*(5), 989–1008. 10.1162/jocn_a_01541 32013688 10.1162/jocn_a_01541 PMC7394318

[CR38] Sali, A. W., Ma, R., Albal, M. S., & Key, J. (2022). The location independence of learned attentional flexibility. *Attention, Perception & Psychophysics,**84*(3), 682–699. 10.3758/s13414-022-02469-4 10.3758/s13414-022-02469-4 35352297

[CR39] Sali, A. W., Bejjani, C., & Egner, T. (2024). Learning cognitive flexibility: Neural substrates of adapting switch-readiness to time-varying demands. *Journal of Cognitive Neuroscience,**36*(2), 377–393. 10.1162/jocn_a_02091 38010299 10.1162/jocn_a_02091 PMC10902878

[CR40] Schneider, D. W. (2016). Perceptual and conceptual priming of cue encoding in task switching. *Journal of Experimental Psychology: Learning, Memory, and Cognition,**42*(7), 1112–1126. 10.1037/xlm0000232 26766605 10.1037/xlm0000232

[CR41] Schneider, D. W., & Logan, G. (2006). Priming cue encoding by manipulating transition frequency in explicitly cued task switching. *Psychonomic Bulletin & Review,**13*(1), 145–151. 10.3758/BF03193826 16724782 10.3758/bf03193826

[CR42] Schneider, D. W., & Logan, G. (2007). Task switching versus cue switching: Using transition cuing to disentangle sequential effects in task-switching performance. *Journal of Experimental Psychology: Learning, Memory, and Cognition,**33*(2), 370–378. 10.1037/0278-7393.33.2.370 17352618 10.1037/0278-7393.33.2.370

[CR43] Singmann, H., Bolker, B., Westfall, J., Aust, F., Ben-Shachar, M. S., Højsgaard, S., ...., & Christensen, R. H. B. (2024). *afex: Analysis of factorial experiments* (Version 1.4-1) [Computer software]. https://cran.r-project.org/web/packages/afex/index.html. Accessed 28 Oct 2024

[CR44] Sternberg, S. (1969). The discovery of processing stages: Extensions of Donders’ method. *Acta Psychologica,**30*, 276–315. 10.1016/0001-6918(69)90055-9

[CR45] Summerfield, C., Egner, T., Greene, M., Koechlin, E., Mangels, J., & Hirsch, J. (2006). Predictive codes for forthcoming perception in the frontal cortex. *Science,**314*(5803), 1311–1314. 10.1126/science.1132028 17124325 10.1126/science.1132028

[CR46] Sun, Z., & Firestone, C. (2020). The Dark Room Problem. *Trends in Cognitive Sciences,**24*(5), 346–348. 10.1016/j.tics.2020.02.006 32298620 10.1016/j.tics.2020.02.006

[CR47] Sutton, R. S., & Barto, A. G. (1998). *Reinforcement learning: An introduction*. Bradford.

[CR48] Theeuwes, J. (1992). Perceptual selectivity for color and form. *Perception & Psychophysics,**51*, 599–606. 1620571 10.3758/bf03211656

[CR49] Townsend, J. T. (1990). Serial vs. parallel processing: Sometimes they look like Tweedledum and Tweedledee but they can (and should) be distinguished. *Psychological Science,**1*(1), 46–54.

[CR50] Townsend, J. T., & Nozawa, G. (1995). Spatio-temporal properties of elementary perception: An investigation of parallel, serial, and coactive theories. *Journal of Mathematical Psychology,**39*(4), 321–359. 10.1006/jmps.1995.1033

[CR51] Vossel, S., Weidner, R., Thiel, C. M., & Fink, G. R. (2009). What is “odd” in Posner’s location-cueing paradigm? Neural responses to unexpected location and feature changes compared. *Journal of Cognitive Neuroscience,**21*(1), 30–41. 10.1162/jocn.2009.21003 18476756 10.1162/jocn.2009.21003

[CR52] Walsh, K. S., McGovern, D. P., Clark, A., & O’Connell, R. G. (2020). Evaluating the neurophysiological evidence for predictive processing as a model of perception. *Annals of the New York Academy of Sciences,**1464*(1), 242–268. 10.1111/nyas.14321 32147856 10.1111/nyas.14321 PMC7187369

[CR53] Wickham, H., Averick, M., Bryan, J., Chang, W., McGowan, L., François, R.,. ...., &Yutani, H. (2019). Welcome to the Tidyverse. *Journal of Open Source Software*, *4*(43), 1686. 10.21105/joss.01686

[CR54] Wilke, C. O. (2024). *cowplot: Streamlined plot theme and plot annotations for “ggplot2”* (Version 1.1.3) [Computer software]. https://cran.r-project.org/web/packages/cowplot/index.html. Accessed 28 Oct 2024

[CR55] Yantis, S., & Jonides, J. (1984). Abrupt visual onsets and selective attention: Evidence from visual search. *Journal of Experimental Psychology*. *Human Perception and Performance,**10*, 601–621. 6238122 10.1037//0096-1523.10.5.601

[CR56] Yashar, A., & Lamy, D. (2013). Temporal position priming: Memory traces of recent experience bias the allocation of attention in time. *Journal of Experimental Psychology: Human Perception and Performance,**39*(5), 1443–1456. 10.1037/a0031231 23339347 10.1037/a0031231

