# Correction to: Serial processing of stimulus identity and shift readiness predictions

**DOI:** 10.3758/s13414-026-03231-w

**Published:** 2026-02-16

**Authors:** Anthony W. Sali, Emily E. Oor

**Affiliations:** Department of Psychology, Wake Forest University, 1834 Wake Forest Road, Winston-Salem, NC 27109 USA

**Correction to** **:** **Atten Percept Psychophys**

10.3758/s13414-025-03137-z

Following publication, we discovered that a single participant completed Experiment 2 twice. In our initial published results, each of these sessions was treated as a unique participant. We have rerun all analyses in Experiment 2 with the second session from this participant excluded, and all conclusions remain the same as in our published paper. A mention of a marginal main effect in the block half analysis should be removed, as it was no longer present at *p* < .10. Additionally, we would like to correct a footnote in our original paper that mistakenly stated that no participants were assigned different groups upon restarting the task. Below, we list the corrections to the footnote and to the numerical values reported in Experiment 2. The footnote, values, tables, and figures have been corrected in the original article.

1. The footnote should read: “In both Experiments 1 and 2, participants were able to restart the task by either returning to the online consent form or refreshing the browser window running the task. In some cases, the participant was randomly assigned to a different stimulus probability condition upon restarting. We have elected to retain these participants since any restarts likely occurred early in the task and would create noise in the data that would only weaken stimulus likelihood learning (Experiments 1 and 2) and cue type recognition (Experiment 2). Here, and in Experiment 2, we do not report individuals who ended the study prior to submitting their data or experienced a technical error.”
2. The demographic information should change as follows. A total of 129 individuals (60 men, 69 women), ranging in age from 18 to 44 years (*M* = 32.8, *SD* = 6.25) completed the task online to the point of successfully submitting their data. A single individual completed Experiment 2 twice due to an error, and we only include data from their first session in all analyses reported here.
3. The final sample size was 95. Those participants excluded for low accuracy (*M* = 32.7, *SD* = 6.14) did not differ in age from those included in the final sample (*M* = 32.9, *SD* = 6.32), *t*(127) = 0.15, *p* = .882.
4. The mean number of participants in each version of the task was 5.94 (*SD* = 3.32) across all potential groups.
5. The following numerical changes apply to the 2 × 2 × 2 repeated-measures ANOVA of response times.
  - Main effect of cue type: *F*(1,94) = 161.87, *p* < .001, η_p_^2^ = .633
  - Main effect of stimulus identity likelihood: *F*(1,94) = 17.57, *p* < .001, η_p_^2^ = .157
  - Main effect of shift likelihood: *F*(1,94) = 3.33, *p* = .071, η_p_^2^ = .034
  - Interaction of cue type by shift likelihood: *F*(1,94) = 216.23, *p* < .001, η_p_^2^ = .697
  - Interaction of cue type by stimulus identity likelihood: *F*(1,94) = 5.78, *p* = .018, η_p_^2^ = .058
  - Interaction of shift likelihood by stimulus identity likelihood: *F*(1,94) = 0.08, *p* = .782, η_p_^2^ < .001
  - Three-way interaction: *F*(1,94) = 0.07, *p* = .791, η_p_^2^ < .001
6. The Bayes factors had the following numerical changes.
  - The Bayes factor for the model that best accounted for the data should be BF_10_ = 3.066×10^44^ with respect to the null model.
  - The interaction of cue type by shift likelihood: BF_inc_ = 1.150×10^23^
  - The interaction of shift likelihood by stimulus identity likelihood: BF_inc_ = 0.130
  - The interaction of cue type by stimulus identity likelihood: BF_inc_ = 1.437
  - The three-way interaction of cue type, shift likelihood, and stimulus identity likelihood, BF_inc_ = 0.172
7. The following numerical changes apply to the 2 × 2 × 2 repeated-measures ANOVA of accuracies.
  - The main effect of stimulus likelihood: *F*(1,94) = 7.09, *p* = .009, η_p_^2^ = .070
  - The main effect of cue type: *F*(1,94) = 2.85, p = .095, η_p_^2^ = .029
  - The interaction of cue type by shift likelihood: *F*(1,94) = 28.56, *p* < .001, η_p_^2^ = .233
  - The statement of other main effects or interactions: *F* values < 1.03, *p* values > .313
8. The following numerical changes apply to the repeated measures ANOVAs testing differences in flexibility for the first and second half of each block.
  - Main effect of cue type, *F*(1,94) = 122.47, *p* < .001, η_p_^2^ = .566
  - Interaction of cue type by shift likelihood: *F*(1,94) = 214.52, *p* < .001, η_p_^2^ = .695
  - The previously reported trend for the main effect of shift likelihood is no longer trending at *p* < .1 and should be deleted.
  - Interaction of shift likelihood by block half: *F*(1,94) = 4.39, *p* = .039, η_p_^2^ = .045
  - Three-way interaction: *F*(1,94) = 15.06, *p* < .001, η_p_^2^ = .138
  - The statement regarding the remaining main effects and interaction: *F* values < 2.70, *p* values > .104
  - In the follow-up ANOVA for the first half of blocks alone, the interaction of cue type by shift likelihood remained significant: *F*(1,94) = 117.78, *p* < .001, η_p_^2^ = .556.

The original article has been corrected.

