# Supplementary material for: Serial processing of stimulus identity and shift readiness predictions
Source: Atten Percept Psychophys. 2025 Aug 5;87(8):2352–69. doi: 10.3758/s13414-025-03137-z (PMC12568794; doi:10.3758/s13414-025-03137-z)
Supplement: Supplementary file 1 — Supplementary file1 (DOCX 24 KB) [file 13414_2025_3137_MOESM1_ESM.docx]

**Supplemental Material**

**Supplemental Table 1.** Bayesian Repeated Measures ANOVA Model Comparison for All Trials in Experiment 1

| Models | P(M) | P(M \| data) | BF_10_ | Error % |
| --- | --- | --- | --- | --- |
| Null Model | .053 | 1.392×10^-38^ | 1.000 |  |
| CueType + ShiftLike + StimLike + CueType×ShiftLike | .053 | 0.500 | 3.591×10^37^ | 4.016 |
| CueType + ShiftLike + StimLike + CueType×ShiftLike + CueType×StimLike + ShiftLike×StimLike + CueType×ShiftLike×StimLike | .053 | 0.284 | 2.042×10^37^ | 5.966 |
| CueType + ShiftLike + StimLike + CueType×ShiftLike + CueType×StimLike | .053 | 0.103 | 7.393×10^36^ | 3.558 |
| CueType + ShiftLike + StimLike + CueType×ShiftLike + ShiftLike×StimLike | .053 | 0.092 | 6.629×10^36^ | 3.544 |
| CueType + ShiftLike + StimLike + CueType×ShiftLike + CueType×StimLike + ShiftLike×StimLike | .053 | 0.020 | 1.470×10^36^ | 4.129 |
| CueType + ShiftLike + CueType×ShiftLike | .053 | 1.962×10^-6^ | 1.409×10^32^ | 3.306 |
| CueType + StimLike | .053 | 2.759×10^-14^ | 1.982×10^24^ | 3.343 |
| CueType + ShiftLike + StimLike | .053 | 1.278×10^-14^ | 9.181×10^23^ | 3.115 |
| CueType + StimLike + CueType×StimLike | .053 | 6.075×10^-15^ | 4.363×10^23^ | 3.817 |
| CueType + ShiftLike + StimLike + CueType×StimLike | .053 | 2.951×10^-15^ | 2.120×10^23^ | 4.351 |
| CueType + ShiftLike + StimLike + ShiftLike×StimLike | .053 | 2.649×10^-15^ | 1.902×10^23^ | 4.328 |
| CueType + ShiftLike + StimLike + CueType×StimLike + ShiftLike×StimLike | .053 | 5.414×10^-16^ | 3.889×10^22^ | 3.825 |
| CueType | .053 | 1.102×10^-19^ | 7.916×10^18^ | 3.024 |
| CueType + ShiftLike | .053 | 5.314×10^-20^ | 3.817×10^18^ | 3.336 |
| StimLike | .053 | 3.294×10^-33^ | 236,619.003 | 3.057 |
| ShiftLike + StimLike | .053 | 1.595×10^-33^ | 114,536.728 | 3.319 |
| ShiftLike + StimLike + ShiftLike×StimLike | .053 | 3.155×10^-34^ | 22,657.868 | 3.731 |
| ShiftLike | .053 | 6.351×10^-39^ | 0.456 | 3.013 |

*Note. Null model accounts for participants and random slopes. P(M) = prior model probability, P(M | data) = posterior model probabilities, BF_10_ = Bayes Factor comparing each model to the null model, error % = estimate of numerical error when calculating Bayes Factors. The models are sorted according to the comparison to the null model. CueType denotes shift vs. hold, while ShiftLike and StimLike denote the shift and stimulus identity likelihoods, respectively.*

**Supplemental Table 2.** Bayesian Repeated Measures ANOVA Model Comparison Excluding Cue Stimulus Repetitions for Experiment 1

| Models | P(M) | P(M \| data) | BF_10_ | Error % |
| --- | --- | --- | --- | --- |
| Null Model | .053 | 8.437×10^-32^ | 1.000 |  |
| CueType + ShiftLike + StimLike + CueType×ShiftLike | .053 | 0.426 | 5.050×10^30^ | 3.859 |
| CueType + ShiftLike + StimLike + CueType×ShiftLike + CueType×StimLike | .053 | 0.390 | 4.623×10^30^ | 2.977 |
| CueType + ShiftLike + StimLike + CueType×ShiftLike + ShiftLike×StimLike | .053 | 0.076 | 9.044×10^29^ | 3.060 |
| CueType + ShiftLike + StimLike + CueType×ShiftLike + CueType×StimLike + ShiftLike×StimLike | .053 | 0.076 | 8.990×10^29^ | 3.941 |
| CueType + ShiftLike + StimLike + CueType×ShiftLike + CueType×StimLike + ShiftLike×StimLike + CueType×ShiftLike×StimLike | .053 | 0.031 | 3.719×10^29^ | 5.432 |
| CueType + ShiftLike + CueType×ShiftLike | .053 | 3.636×10^-4^ | 4.309×10^27^ | 2.879 |
| CueType + StimLike | .053 | 2.394×10^-11^ | 2.838×10^20^ | 2.945 |
| CueType + StimLike + CueType×StimLike | .053 | 2.331×10^-11^ | 2.763×10^20^ | 3.186 |
| CueType + ShiftLike + StimLike + CueType×StimLike | .053 | 1.479×10^-11^ | 1.753×10^20^ | 4.093 |
| CueType + ShiftLike + StimLike | .053 | 1.458×10^-11^ | 1.728×10^20^ | 2.713 |
| CueType + ShiftLike + StimLike + ShiftLike×StimLike | .053 | 2.915×10^-12^ | 3.455×10^19^ | 4.137 |
| CueType + ShiftLike + StimLike + CueType×StimLike + ShiftLike×StimLike | .053 | 2.648×10^-12^ | 3.138×10^19^ | 3.188 |
| CueType | .053 | 2.072×10^-14^ | 2.456×10^17^ | 2.564 |
| CueType×ShiftLike | .053 | 1.307×10^-14^ | 1.550×10^17^ | 2.932 |
| StimLike | .053 | 9.363×10^-29^ | 1,109.792 | 2.600 |
| ShiftLike + StimLike | .053 | 5.924×10^-29^ | 702.126 | 2.928 |
| ShiftLike + StimLike + ShiftLike×StimLike | .053 | 1.137×10^-29^ | 134.812 | 3.219 |
| ShiftLike | .053 | 5.113×10^-32^ | 0.606 | 2.537 |

*Note. Null model accounts for participants and random slopes. P(M) = prior model probability, P(M | data) = posterior model probabilities, BF_10_ = Bayes Factor comparing each model to the null model, error % = estimate of numerical error when calculating Bayes Factors. The models are sorted according to the comparison to the null model. CueType denotes shift vs. hold, while ShiftLike and StimLike denote the shift and stimulus identity likelihoods, respectively.*

**Supplemental Table 3.** Bayesian Repeated Measures ANOVA Model Comparison for Experiment 2

| Models | P(M) | P(M \| data) | BF_10_ | Error % |
| --- | --- | --- | --- | --- |
| Null Model | .053 | 1.543×10^-45^ | 1.000 |  |
| CueType + ShiftLike + StimLike + CueType×ShiftLike + CueType×StimLike | .053 | 0.485 | 3.147×10^44^ | 2.244 |
| CueType + ShiftLike + StimLike + CueType×ShiftLike | .053 | 0.382 | 2.478×10^44^ | 3.281 |
| CueType + ShiftLike + StimLike + CueType×ShiftLike + CueType×StimLike + ShiftLike×StimLike | .053 | 0.068 | 4.422×10^43^ | 2.430 |
| CueType + ShiftLike + StimLike + CueType×ShiftLike + ShiftLike×StimLike | .053 | 0.051 | 3.294×10^43^ | 2.321 |
| CueType + ShiftLike + StimLike + CueType×ShiftLike + CueType×StimLike + ShiftLike×StimLike + CueType×ShiftLike×StimLike | .053 | 0.011 | 7.360×10^42^ | 4.440 |
| CueType + ShiftLike + CueType×ShiftLike | .053 | 0.002 | 1.309×10^42^ | 2.236 |
| CueType + StimLike + CueType×StimLike | .053 | 2.277×10^-24^ | 1.476×10^21^ | 2.296 |
| CueType + ShiftLike + StimLike + CueType×StimLike | .053 | 1.931×10^-24^ | 1.252×10^21^ | 3.382 |
| CueType + StimLike | .053 | 1.701×10^-24^ | 1.103×10^21^ | 2.177 |
| CueType + ShiftLike + StimLike | .053 | 1.401×10^-24^ | 9.085×10^20^ | 2.070 |
| CueType + ShiftLike + StimLike + CueType×StimLike + ShiftLike×StimLike | .053 | 2.565×10^-25^ | 1.663×10^20^ | 2.243 |
| CueType + ShiftLike + StimLike + ShiftLike×StimLike | .053 | 2.021×10^-25^ | 1.310×10^20^ | 3.382 |
| CueType | .053 | 9.010×10^-27^ | 5.841×10^18^ | 1.756 |
| CueType + ShiftLike | .053 | 7.608×10^-27^ | 4.932×10^18^ | 2.162 |
| StimLike | .053 | 2.869×10^-43^ | 185.977 | 1.758 |
| ShiftLike + StimLike | .053 | 2.434×10^-43^ | 157.816 | 2.164 |
| ShiftLike + StimLike + ShiftLike×StimLike | .053 | 3.425×10^-44^ | 22.201 | 2.299 |
| ShiftLike | .053 | 1.282×10^-45^ | 0.831 | 1.748 |

*Note. Null model accounts for participants and random slopes. P(M) = prior model probability, P(M | data) = posterior model probabilities, BF_10_ = Bayes Factor comparing each model to the null model, error % = estimate of numerical error when calculating Bayes Factors. The models are sorted according to the comparison to the null model. CueType denotes shift vs. hold, while ShiftLike and StimLike denote the shift and stimulus identity likelihoods, respectively.*
